# A Selective Small‐Molecule c‐Myc Degrader Potently Regresses Lethal c‐Myc Overexpressing Tumors

**DOI:** 10.1002/advs.202104344

**Published:** 2022-01-20

**Authors:** Ying Xu, Qingfeng Yu, Ping Wang, Zhaoxing Wu, Lei Zhang, Shuigao Wu, Mengyuan Li, Bowen Wu, Hongzhi Li, Haifeng Zhuang, Xuzhao Zhang, Yu Huang, Xiaoxian Gan, Rongzhen Xu

**Affiliations:** ^1^ Department of Hematology and Cancer Institute (Key Laboratory of Cancer Prevention and Intervention China National Ministry of Education Key Laboratory of Molecular Biology in Medical Sciences The Second Affiliated Hospital Zhejiang University School of Medicine Hangzhou 310009 China; ^2^ Weben Pharmaceuticals Hangzhou 310051 China; ^3^ Department of Molecular Medicine Beckman Research Institute City of Hope National Medical Center Duarte CA 91010 USA; ^4^ Department of Hematology the First Affiliated Hospital of Zhejiang Chinese Medical University Hangzhou 310009 China; ^5^ Academy of Chinese Medical Sciences Zhejiang Chinese Medical University Hangzhou 310053 China; ^6^ Institute of Hematology Zhejiang University Hangzhou 310009 China

**Keywords:** cancer, c‐Myc oncoprotein, proteolysis targeting molecule, targeted therapy, WBC100

## Abstract

*MYC* oncogene is involved in the majority of human cancers and is often associated with poor outcomes, rendering it an extraordinarily desirable target, but therapeutic targeting of c‐Myc protein has been a challenge for >30 years. Here, WBC100, a novel oral active molecule glue that selectively degrades c‐Myc protein over other proteins and potently kills c‐Myc overexpressing cancer cells is reported. WBC100 targets the nuclear localization signal 1 (NLS1)–Basic–nuclear localization signal 2 (NLS2) region of c‐Myc and induces c‐Myc protein degradation through ubiquitin E3 ligase CHIP mediated 26S proteasome pathway, leading to apoptosis of cancer cells. In vivo, WBC100 potently regresses multiple lethal c‐Myc overexpressing tumors such as acute myeloid leukemia, pancreatic, and gastric cancers with good tolerability in multiple xenograft mouse models. Identification of the NLS1–Basic–NLS2 region as a druggable pocket for targeting the “undruggable” c‐Myc protein and that single‐agent WBC100 potently regresses c‐Myc overexpressing tumors through selective c‐Myc proteolysis opens new perspectives for pharmacologically intervening c‐Myc in human cancers.

## Graphical abstract

This study identifies the NLS1–Basic–NLS2 domain as a druggable pocket for directly targeting c‐Myc protein. As a novel potent and selective c‐Myc inhibitor, WBC100 directly targets nuclear c‐Myc and E3 ligase CHIP for proteasome‐dependent degradation. Importantly, WBC100 regresses c‐Myc overexpressing acute myeloid leukemia, pancreatic, and gastric cancers in mouse models.

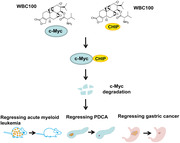

## 1 Introduction

Cancer is one of the leading causes of death worldwide, and both the incidence and prevalence of cancer continue to increase.^[^ ^1^ ^]^ The *MYC* oncogene, a “master driver” in human cancers, is aberrantly activated in numerous hematological malignancies including leukemia,^[^ ^2^, ^3^ ^]^ lymphoma^[^ ^4^, ^5^ ^]^ and multiple myeloma,^[^ ^6^ ^]^ and a wide range of solid tumors such as pancreatic ductal adenocarcinoma (PDAC),^[^ ^7^, ^8^ ^]^ brain cancer,^[^ ^9^, ^10^ ^]^ non‐small‐cell lung cancer (NSCLC)^[^ ^11^, ^12^ ^]^ and small‐cell lung cancer (SCLC),^[^ ^13^, ^14^ ^]^ liver cancer,^[^ ^15^, ^16^ ^]^ prostate cancer,^[^ ^17^, ^18^ ^]^ etc. More importantly, c‐Myc hyperactivation is often associated with aggressive, drug resistance and poor clinical outcome.^[^ ^19^, ^20^, ^21^ ^]^ Intriguingly, genetic studies have shown that suppressing c‐Myc activation causes rapid tumor regression by inhibiting cell proliferation, inducing senescence and apoptosis, and remodeling tumor microenvironment in animal models.^[^ ^22^ ^]^ Similarly, depletion of c‐Myc transcriptional activity causes the eradication of oncogenic K‐RAS‐driven lung adenocarcinoma in mice without serious side effects.^[^ ^23^ ^]^ Clearly, targeting c‐Myc represents an important milestone in the era of molecular targeted therapy in human cancers. Despite tremendous effort to develop a clinically viable strategy for effectively treating c‐Myc overexpressing tumors, no drug‐like c‐Myc inhibitors have been developed yet for more than three decades.^[^ ^24^ ^]^ c‐Myc protein has been considered to be “undruggable” due to its helix‐loop‐helix topology lacking druggable domains and nuclear localization within cancer cells, making it difficult to directly target c‐Myc with traditional small‐molecule inhibitors.^[^ ^25^, ^26^, ^27^, ^28^ ^]^ Previous studies reported several small‐molecule c‐Myc protein inhibitors, which could be generally classified into two classes. One of them could directly bind to c‐Myc protein and prevents Myc‐Max heterodimerization, such as 10074‐G5, 10058‐F4, and 10074‐A4,^[^ ^29^, ^30^, ^31^ ^]^ which could bind to the bHLHZip domain of c‐Myc but did not show potent antitumor activity in vivo.^[^ ^32^, ^33^ ^]^ The other could perturb the heterodimer's interaction with its target DNA sequence without causing loss of the heterodimerization, such as synthetic *α*‐helix mimetics.^[^ ^34^ ^]^

Previous studies showed that triptolide (TPL), a natural small molecule, exhibits potently cytocidal action against a subset of human cancer cells and induced rapid reduction of c‐Myc protein in sensitive cancer cells.^[^ ^35^, ^36^, ^37^ ^]^ Triptolide was considered a promising anticancer agent, but its clinical use is largely limited due to poor aqueous solubility, dose‐limited toxicity, and the uncertainty of its antitumor targets. To solve these issues, we designed a panel of derivatives of triptolide. Among them, a novel water‐soluble analog of triptolide, 14‐dextrorotary‐valyl‐triptolide (WBC100), was the most promising one that was also well tolerated by mice. We then used it as a probe to identify its targets for antitumor activity and systematically evaluated its antitumor activity in vitro and in vivo. We found that cancer cells with high expression of c‐Myc were more sensitive compared to those with low c‐Myc levels. Therefore, we speculated that c‐Myc may play an important role in the antitumor activity of WBC100.

Here, we characterize WBC100 as an oral active molecule glue that selectively induces c‐Myc protein degradation through ubiquitin E3 ligase CHIP mediated 26S proteasome pathway and potently regresses established c‐Myc overexpressing tumors with good tolerability in multiple xenograft mouse models.

## 2 Result

### 2.1 WBC100 Preferentially Kills c‐Myc Overexpressing Cancer Cells In Vitro

WBC100 is a novel small molecule, and its synthesis and characterization are available in the supplementary data and patent application (US10238623). The structure and formula of WBC100 and its major metabolite are shown in the Figure S1A,B (Supporting Information). The purity of WBC100 in this study was 99.5% as determined by high‐performance liquid chromatography (HPLC). WBC100 is water‐soluble at acidic pH levels (<pH 5.0).

To investigate whether WBC100 selectively kills c‐Myc overexpressing cancer cells, we treated c‐Myc overexpressing human cancer cell lines Mia‐paca2 (PDAC), H9 (T‐cell lymphoma), and MOLM‐13 (acute myeloid leukemia) as well as c‐Myc‐low normal human cell lines L02 (liver), MRC‐5 (lung) and WI38 (lung) with WBC100 at various concentrations for 72 h. We found that the IC50 values of WBC100 for Mia‐paca2, H9, and MOLM‐13 were 61 × 10^−9^, 17 × 10^−9^, and 16 × 10^−9^ m, respectively, whereas the IC50 values for normal cell lines L02, MRC‐5 and WI38 were 2205 × 10^−9^, 151 × 10^−9^, and 570 × 10^−9^ m, respectively (Figure S1C,D, Supporting Information). c‐Myc‐low normal peripheral blood cells were also relatively insensitive to WBC100 (IC_50_ >150 × 10^−9^ m) (Figure S1E,F, Supporting Information). These results suggest that WBC100 preferentially kills c‐Myc overexpressing cancer cells.

**Figure 1:**
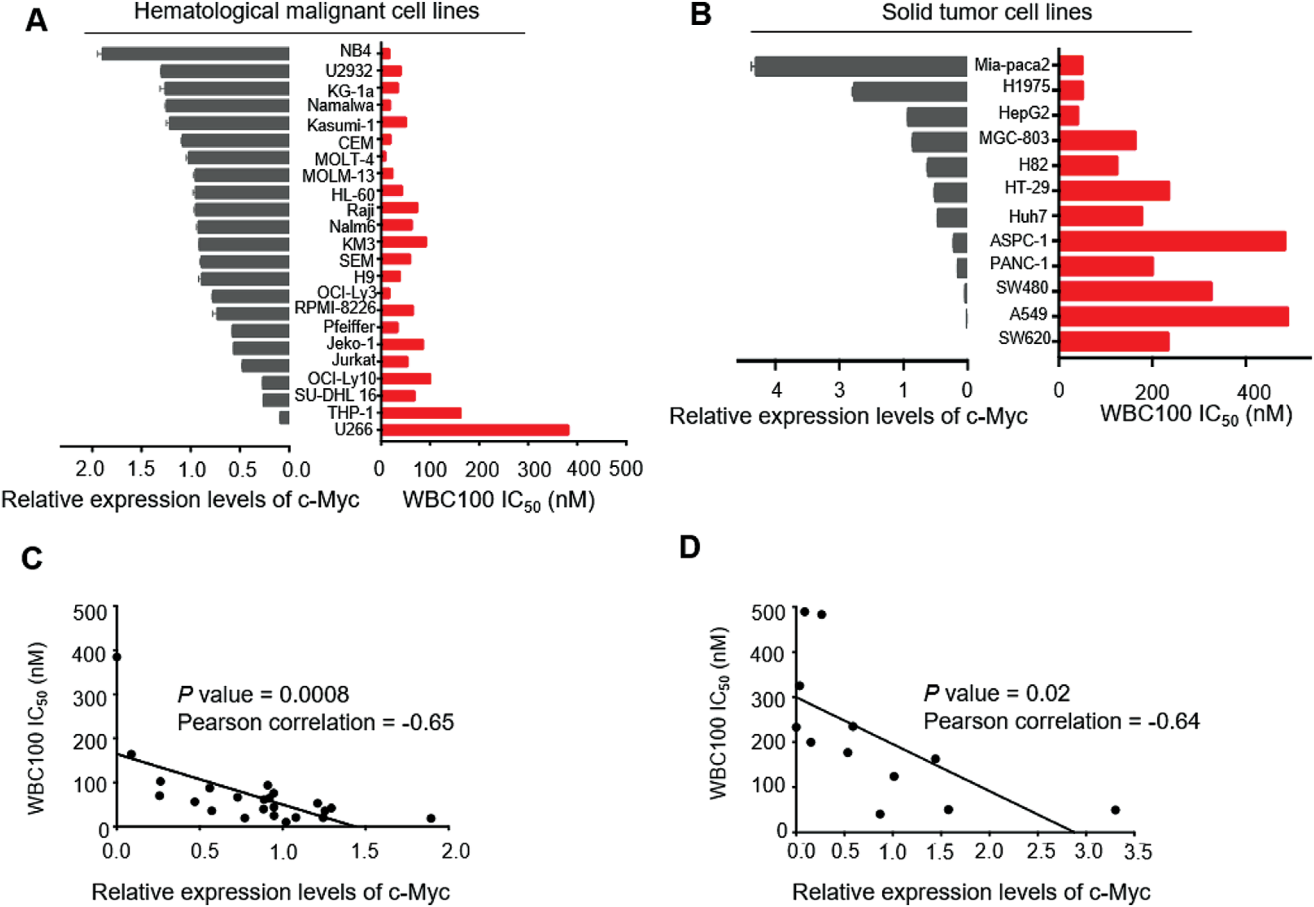
WBC100 preferentially kills c‐Myc overexpressing cancer cells in vitro. A) Western blot analyses of c‐Myc levels (grey) and MTT analyses of IC50 values of WBC100 (red) in 23 various hematological malignant cell lines, including 11 leukemia, 9 lymphoma, and 3 multiple myeloma cell lines. B) Western blot analyses of c‐Myc levels (grey) and MTT analyses of IC50 values of WBC100 (red) in 12 various solid tumor cell lines, including pancreatic ductal adenocarcinoma (PDAC), lung cancer (NSCLC), liver cancer, gastric cancer, and colon cancer cell lines. C,D) Correlation analyses between WBC100 antitumor activity and c‐Myc levels in various C) hematological malignant cell lines (*n* = 23) and D) solid tumor cell lines (*n* = 12). The Pearson correlation coefficient and *p*‐value calculated by linear regression and correlation analysis using GraphPad are shown. Mean ± s.d. of relative expression levels of c‐Myc were shown (*n* = 3). Statistical significance was determined by a two‐tailed *t*‐test.

We next examined the correlation between c‐Myc levels and WBC100 antitumor activity using more tumor cell lines. We analyzed c‐Myc protein levels in a panel of different hematological malignant cell lines, including 11 leukemia, 9 lymphoma, 3 multiple myeloma, and 12 various solid tumor cell lines using western blot. We found that 82.61% (19/23) of hematological malignant cell lines and 58.33% (7/12) of various solid tumor cell lines tested expressed high levels of c‐Myc protein (**Figure** **1**A,B). We then evaluated in vitro WBC100 cytocidal action against these tumor cell lines using MTT assay. Out of 23 various hematological malignant cell lines, 11 hematological cell lines were highly sensitive to WBC100 (IC_50_ ≤ 50 × 10^−9^ m), 9 were medium sensitive (50 × 10^−9^ m < IC_50_ < 100 × 10^−9^ m) and 3 were low sensitive (IC_50_ ≥ 100 × 10^−9^ m) (Figure 1A). These observations indicate that most c‐Myc overexpressing hematological tumor cell lines are sensitive to WBC100. Similarly, out of 12 solid tumor cell lines, 3 cell lines with high c‐Myc protein level were highly sensitive to WBC100 (IC_50_ ≤ 50 × 10^−9^ m) and 3 cell lines with a medium level of c‐Myc protein were demonstrated moderate sensitivity to WBC100 (100 × 10^−9^ m < IC_50_< 200 × 10^−9^ m), whereas 6 tumor cells with low levels of c‐Myc were low sensitive (IC_50_ >200 × 10^−9^ m) (Figure 1B). We next analyzed the correlation between the c‐Myc level and killing activity of WBC100 in various hematological tumor cell lines and found that the killing activity of WBC100 against tumor cells correlated with the c‐Myc protein level (*r* = −0.65, *p* = 0.0008) (Figure 1C) but did not correlate with the nuclear XPB protein level (*r* = −0.05, *p* = 0.83) (Figure S1G,I, Supporting Information). Similar results were observed in solid tumor cell lines (Figure 1D and Figure S1H,J, Supporting Information). To confirm whether c‐Myc is a target of WBC100, WBC100 was chemically linked to biotin (WBC100‐biotin) as a molecular probe. c‐Myc overexpressing cell lysates were incubated with WBC100‐biotin, and proteins were pull‐down with streptavidin‐conjugated beads, isolated by SDS‐PAGE and followed by silver staining and mass spectrometry (MS) analysis. Six proteins were identified, including Hsp70, c‐Myc, E3 ubiquitin ligase CHIP, prohibitin‐1 (PHB1), Peroxiredoxin6 (Prdx6), and Peroxiredoxin1 (Prdx1) in the cellular protein sample enriched by WBC100‐biotin, but not by biotin (Figure S2, Supporting Information). Interestingly, c‐Myc is a client protein of E3 ubiquitin ligase CHIP for proteolysis,^[^ ^38^ ^]^ whereas PHB1 and Prdx1 have been shown to interact with c‐Myc as chaperones.^[^ ^39^, ^40^ ^]^ These results suggest that both c‐Myc and CHIP are potential targets of WBC100.

To determine whether there is a direct relationship of c‐Myc level with WBC100 response, we examined the effect of c‐Myc overexpression (c‐Myc‐OE) using lentivirus transfection in c‐Myc‐low PANC‐1 and A549 cancer cells with resistance to WBC100. We observed that overexpression of c‐Myc protein conferred two‐ to fourfold increase in sensitivity to WBC100 (Figure S3A–F, Supporting Information). In contrast to c‐Myc‐OE, knockdown of endogenous c‐Myc protein (c‐Myc‐KD) using lentiviral transfection in Mia‐paca2 cells conferred 1.8‐fold decreases in sensitivity to WBC100 (Figure S3G–I, Supporting Information). These results suggest a close relationship of c‐Myc levels with WBC100 response.

### 2.2 WBC100 Accumulates in the Nucleus and Induces Apoptosis of c‐Myc Overexpressing Cancer Cells

One of the challenges for targeting c‐Myc is its nuclear localization. To address whether WBC100 could overcome this obstacle, WBC100 was chemically linked to FITC (WBC100‐FITC). We treated c‐Myc overexpressing MOLM‐13 and c‐Myc‐low A549 cells using WBC100‐FITC at 50 × 10^−9^ m concentration for 24 h and investigated the subcellular distribution of WBC100‐FITC under confocal laser scanning microscope. We observed that WBC100‐FITC was enriched in the nucleus of c‐Myc overexpressing MOLM‐13 cells (Figure S4A, Supporting Information). However, no obvious nuclear accumulation of WBC100‐FITC was observed in c‐Myc‐low A549 cells. To further confirm these observations, we determined the extent of WBC100‐FITC of cells by Flow cytometry (FCM) and found that WBC100‐FITC level was higher in c‐Myc overexpressing MOLM‐13 cells than that in c‐Myc‐low A549 cells (45.6% vs 1.9%) (Figure S4B, Supporting Information). To determine whether WBC100 induces apoptosis of c‐Myc overexpressing cancer cells, we treated MOLM‐13 cells with WBC100 at different concentrations and then collected cells for apoptosis analysis by FCM. We found that WBC100 treatment caused significant apoptosis of MOLM‐13 cells in dose‐ and time‐dependent manners (Figure S4C–E, Supporting Information). Western blot analysis showed that apoptosis‐related molecules cleaved caspase3 and cleaved PARP were observed in MOLM‐13 cells (Figure S4F, Supporting Information) after treatment with WBC100‐FITC for 24 h. To investigate whether WBC100‐mediated apoptosis is c‐Myc dependent, we carried out overexpression or knockdown of c‐Myc with tumor cells and evaluated the effects of c‐Myc expression on apoptotic proteins cleaved PARP and cleaved caspase3 with Western blot. Compared to control cells, c‐Myc‐OE increased cleaved PARP and cleaved caspase3 levels in dose‐ and time‐dependent manners in A549 cells (Figure S4G, Supporting Information). In contrast, c‐Myc‐KD decreased cleaved PARP and cleaved caspase3 levels in Mia‐paca2 cells in dose‐and time‐dependent manners (Figure S4H, Supporting Information). These results indicate that WBC100 could penetrate the nucleus and induces apoptosis of cancer cells in a c‐Myc‐dependent way.

### 2.3 WBC100 Directly Binds to the NLS1–Basic–NLS2 Region of c‐Myc Protein

**Figure 2:**
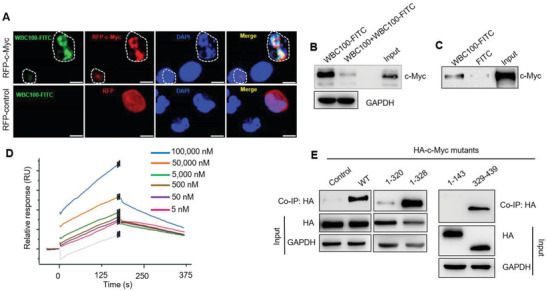
WBC100 directly binds to c‐Myc protein. A) Colocalization analysis of WBC100‐FITC (green) with nuclear RFP‐c‐Myc protein (red) in HEK293T cells after treatment 10 × 10^−6^ m WBC100‐FITC for 24 h. WBC100‐FITC (green) was colocalized with c‐Myc protein (red) and induced apoptosis (circled) of c‐Myc overexpressing cells. RFP: red fluoresce protein. Scale bars: 10 µm. B) Competition pull‐down assay of cellular c‐Myc protein by WBC100‐FITC. Cancer cellular c‐Myc protein was precipitated by WBC100‐FITC and preincubation of excessive unlabeled WBC100 reduced the formation of WBC100‐c‐Myc complexes. C) Western blot analysis for recombinant c‐Myc protein after WBC100‐FITC or FITC (negative control) pull‐down assay. D) Surface plasmon resonance (SPR) results showed the binding of WBC100 to c‐Myc protein in a dose‐dependent manner. E) Binding site mapping of WBC100‐FITC to c‐Myc protein using HA‐tagged c‐Myc (HA‐c‐Myc) protein mutants and co‐IP assay. WT: wild‐type protein.

To reveal whether WBC100 binds to nuclear c‐Myc protein in cells, we expressed c‐Myc that was fused to a red fluorescent protein (RFP‐c‐Myc) using HEK293T cells and then treated the cells with WBC100‐FITC for 24 h. We observed that WBC100‐FITC (green) specifically co‐localized with RFP‐c‐Myc (red) in the nuclei of cells (**Figure** **2**A). In contrast, no colocalization between WBC100‐FITC and RFP was observed in control cells (Figure 2A).

To verify whether WBC100 specifically interacts with c‐Myc protein in cells, we next performed a competitive affinity pull‐down assay using WBC100‐FITC, FITC antibody‐bound magnetic beads, and c‐Myc overexpressing cancer cellular proteins. Unlabeled WBC100 was used as a binding competitor. c‐Myc overexpressing cancer cellular proteins were incubated with WBC100‐FITC in the absence or presence of excess unlabeled WBC100 and the complex then was precipitated with FITC antibody‐bound magnetic beads for western blot analysis. We observed that c‐Myc protein was precipitated by WBC100‐FITC and the binding of WBC100‐FITC to c‐Myc protein was sensitive to competition by excess unlabeled WBC100 (Figure 2B), suggesting that WBC100‐FITC could specifically bind to c‐Myc protein. To further confirm these results, we performed a pull‐down assay using purified recombinant c‐Myc protein. Similarly, the purified c‐Myc protein was precipitated by WBC100‐FITC (Figure 2C), supporting the direct binding of WBC100‐FITC to c‐Myc protein. Moreover, we performed direct binding assay of WBC100 with c‐Myc protein on biosensor chip by surface plasmon resonance (SPR) and found that WBC100 bind to the c‐Myc protein on biosensor chip in a dose‐dependent manner (Ka (1/ms) = 1.07e+01, kd(1/s) = 3.47e‐03, KD = 7.09E‐6 м) (Figure 2D). These results together indicate that WBC100 directly binds to c‐Myc protein.

To reveal the potential binding sites of c‐Myc protein for WBC100, WBC100‐FITC was used. We constructed a series of HA‐tagged recombinant c‐Myc deletion mutants spanning the full‐length c‐Myc protein and then determined the binding ability of WBC100‐FITC to these mutants in HEK293T cells. As expected, we observed that the full‐length c‐Myc protein (WT‐c‐Myc) in the HEK293T cell lysates was precipitated by WBC100‐FITC (Figure 2E). Interestingly, we found that WBC100‐FITC strongly interacted with the mutants c‐Myc1‐328 and c‐Myc329‐439, but not other deletion mutants: c‐Myc1‐143 (transcription activation domain, TAD), and c‐Myc1‐320 (c‐Myc‐nick domain) (Figure 2E). The strong binding of WBC100‐FITC to mutants c‐Myc1‐328 and c‐Myc329‐439 (containing NLS1, Basic, and NLS2 domains), suggests that the NLS1–Basic–NLS2 region of c‐Myc protein might be essential for WBC100 binding.

**Figure 3:**
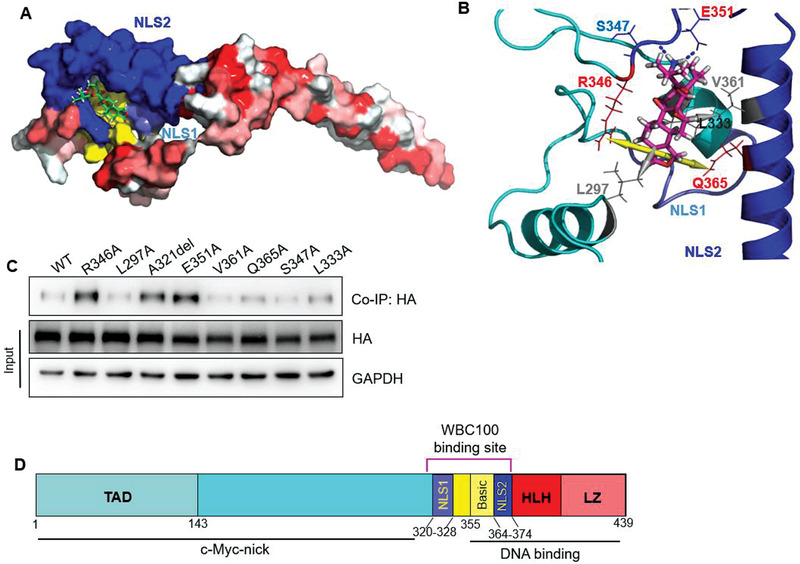
Binding model of WBC100 to c‐Myc protein. A) WBC100 compound (Green) contacts NLS2, Basic and NLS1 motifs in Docking Model. The NLS1, Basic, and NLS2 motifs are colored as light‐blue, yellow, and blue, respectively. B) Interaction networks of WBC100 (pink) in complex with c‐Myc in Docking Model. The WBC100 compound forms two hydrogen bonds (shown as blue dots) with S347/E351, two cation‐*π* interactions with R346 and Q365 (yellow arrows), and a hydrophobic interaction network (grey arrows) with L297/L333/V361. The NLS1, Basic, and NLS2 motifs are colored as light‐blue, yellow, and blue, respectively. C) Binding site mapping of WBC100‐FITC to c‐Myc protein using HA‐c‐Myc protein mutants and competitive co‐IP assay. WT: wild‐type protein. D) Schematic diagram of WBC100 binding to the NLS1–Basic–NLS2 domain of c‐Myc protein.

To further confirm the above observations, we built a docking model of human c‐Myc with WBC100. As shown in our model, c‐Myc forms homodimer,^[^ ^41^ ^]^ or heterodimer with Max protein,^[^ ^31^, ^42^ ^]^ or bound to DNA molecule (Figure S5A, Supporting Information).^[^ ^41^ ^]^ The homology model of monomer c‐Myc in range of 289–439 a.a. was built by merging its two homology models, i.e., the model of 289–378 a.a. region by using eIF3c (PDB id 4u1c, heterodimer for eIF3c) since so far there is no X‐ray structure for the NLS1 region yet and eIF3c shows similarity to c‐Myc with 27% sequence identity (Figure S5B, Supporting Information), and that of 350–439 a.a. region by using OmoMyc structure (PDB id 5i50, homodimer bound to DNA). DNA molecule was removed from the model. We used PDB 5i50 instead of Myc‐Max heterodimer (PDB 1nkp)^[^ ^43^ ^]^ as partial model structure since the two structures are almost identical and PDB 5i50 is longer than PDB 1nkp, which can make a better overall model (Figure S5C, Supporting Information). However, we also found that WBC100 binds at a region that is not the dimer interaction area (Figure S5D, Supporting Information). Therefore, we only displayed the binding of WBC100 on one monomer (**Figure** **3**A). The overlapping region (357–379 a.a. helix) of the two structures was used for structural alignment and model merging, as a template on Swiss Model server.^[^ ^44^ ^]^ The best binding pockets for WBC100 were predicted by implementing our in‐house developed All‐Around Docking (AAD) method.^[^ ^45^ ^]^ AAD allows a small molecule to dock on the whole surface of a protein to search for the best docking pocket. We observed that WBC100 preferred to bind the NLS1–Basic–NLS2 region, as shown in Figure 3A. The WBC100 can form hydrogen bonds, hydrophobic interaction, and cation‐*π* interaction with c‐Myc protein. As displayed in Figure 3B, WBC100 forms two hydrogen bonds (shown as blue dots) with S347/E351, two cation‐*π* interactions with R346 and Q365 (yellow arrows), and hydrophobic interaction network (grey arrows) with L297/L333/V361. The NLS1, Basic and NLS2 motifs are colored as light‐blue, yellow and blue, respectively.

To verify whether WBC100 is clamped by these amino acid residues, we constructed 8 mutants of c‐Myc: L297A, A321del, L333A, R346A, S347A, E351A, V361A, and Q365A using site‐directed mutagenesis. We next transfected HEK293T cells with these c‐Myc mutants for 48 h and then extracted cellular proteins for binding assay of WBC100‐FITC with these c‐Myc mutants. To prevent nonspecific binding, we carried out competitive pull‐down assays using unlabeled WBC100 as a specific binding competitor. In brief, c‐Myc mutant cellular proteins were pre‐incubated with unlabeled WBC100 at 4 ℃ overnight and then incubated with WBC100‐FITC at 4 ℃ overnight. The complex of c‐Myc mutants with WBC100‐FITC was precipitated with FITC antibody‐bound magnetic beads for western blot analysis. Western blot results showed that binding of WT‐c‐Myc, L297A, V361A, and S347A with FITC antibody‐bound magnetic beads were nearly completely blocked by unlabeled WBC100, whereas the binding blocking of c‐Myc mutants R346A, A321del, E351A, Q365A, and L333A by unlabeled WBC100 was attenuated, especially R346A, A321del, and E351A, suggesting that c‐Myc amino acids at R346, A321, E351, Q365, and L333 might be important for WBC100 binding (Figure 3C). These results are consistent with that of the docking model, further confirming that the NLS1–Basic–NLS2 region is the binding site of WBC100 (Figure 3D). To test whether these mutations affect c‐Myc function, we compared the effects of c‐Myc mutants R346A, A321del, and E351A with wild‐type c‐Myc on cell proliferation in c‐Myc‐low A549 cells. We observed that c‐Myc mutants R346A and E351A showed a decreased cell proliferation ability as compared with wild‐type c‐Myc protein (Figure S6A, Supporting Information). To further determine if WBC100‐mediated antitumor activity was affected by these mutations, we next compared the effects of c‐Myc mutants R346A, A321del, and E351A on WBC100‐mediated antitumor activity in c‐Myc‐low A549 cells. Consistently, we found that c‐Myc mutants R346A, A321del, and E351A decreased WBC100‐mediated antitumor activity as compared with wild‐type c‐Myc (Figure S6B,C, Supporting Information).

### 2.4 WBC100 Selectively Induces Degradation of c‐Myc Protein through E3 Ubiquitin Ligase CHIP Mediated 26S Proteasome Pathway

**Figure 4:**
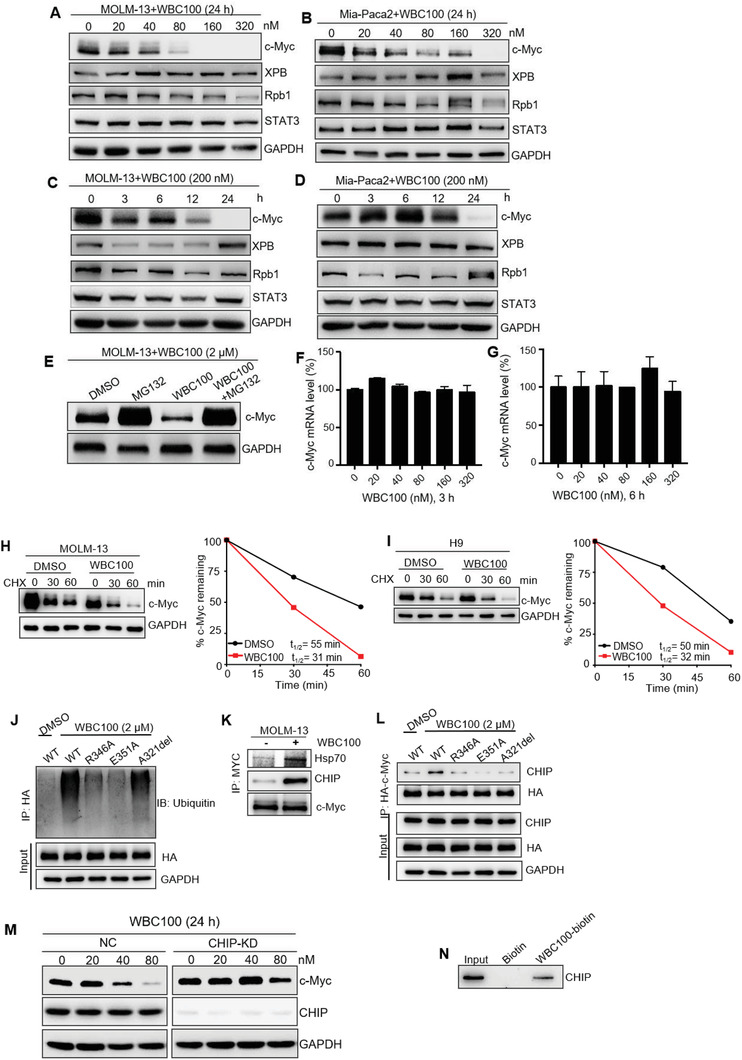
WBC100 selectively induces c‐Myc protein degradation mediated by ubiquitin E3 ligase CHIP. c‐Myc, XPB, Rpb1, and STAT3 levels in A) MOLM‐13 and B) Mia‐paca2 cells treated with WBC100 for 24 h, assessed by western blot. Western blot analysis for c‐Myc, XPB, Rpb1, and STAT3 in WBC100 treated C) MOLM‐13 and D) Mia‐paca2 cells at indicated time points. E) Treatment with a proteasome inhibitor MG‐132 (1 × 10^−6^ m) attenuated WBC100‐induced c‐Myc degradation. c‐Myc levels were determined by western blots at 6 h after WBC100 and/or MG‐132 (1 × 10^−6^ m) treatment. F,G) c‐Myc mRNA levels in MOLM‐13 cells were determined using RT‐qPCR at 3, 6 h after WBC100 treatment (*n* = 2). H) MOLM‐13 and I) H9 cells were pretreated with WBC100 (2 × 10^−6^ m) or DMSO for 6 h, followed by cycloheximide (CHX, 25 × 10^−6^ m) treatment. Cells were harvested at the indicated time points and c‐Myc levels were determined by western blot. c‐Myc protein degradation kinetic curves based on the quantification of c‐Myc levels. J) HEK293T cells were transfected with HA‐tagged wild‐type c‐Myc (WT) or HA‐tagged mutants of c‐Myc (R346A, E351A, A321del). At 48 h after transfection, cells were treated with WBC100 (2 × 10^−6^ m) or DMSO in the presence of MG‐132 (10 × 10^−6^ m) for 4 h. Ubiquitinated HA‐tagged proteins were pulled down by immunoprecipitation with anti‐HA magnetic beads and assessed by western blot using ubiquitin‐specific monoclonal antibodies. K) MOLM‐13 cells were treated with WBC100 (2 × 10^−6^ m) or DMSO in the presence of MG‐132 (10 × 10^−6^ m) for 4 h. Western blot showed the levels of CHIP, Hsp70 co‐immunoprecipitated with c‐Myc in MOLM‐13 cells. L) HEK293T cells were transfected with HA‐c‐Myc and HA‐c‐Myc mutants. At 48 h after transfection, cells were treated with DMSO or WBC100 (2 × 10^−6^ m) in the presence of MG‐132 (10 × 10^−6^ m) for 4 h. Western blot showed the levels of CHIP co‐immunoprecipitated with HA‐c‐Myc and its HA‐tagged mutants. M) Mia‐paca2 cells were transfected with CHIP siRNA (100 × 10^−9^ m) or nontargeting control (NC)‐siRNA using Lipofectamine 3000. At 48 h after transfection, cells were treated with WBC100. Western blot analysis of c‐Myc and CHIP levels in CHIP‐KD and NC cells treated with WBC100 for 24 h. CHIP‐KD: CHIP knockdown. N) Western blot analysis for recombinant CHIP protein after WBC100‐biotin or biotin (negative control) pull‐down assay. All error bars represent means ± s.d. Statistical significance was determined by the two‐tailed *t*‐test. *p < 0.05; **p < 0.01; ***p < 0.001; ****p < 0.0001.

To determine whether WBC100 could affect c‐Myc stability in cancer cells, we examined its effects on c‐Myc and other nuclear proteins including XPB, the largest subunit of RNA polymerase II (Rpb1), and STAT3 in various cancer cells, respectively, using western blotting. We observed that WBC100 decreased c‐Myc protein levels in MOLM‐13 cells and Mia‐paca2 cells in a dose‐dependent manner, but had no obvious impact on XPB, Rpb1, and STAT3 (**Figure** **4**A,B). The time course studies showed that incubation with WBC100 led to a reduction in c‐Myc protein levels in a time‐dependent manner, reaching a maximum effect at 24 h after treatment (Figure 4C,D). Furthermore, we found that co‐treatment with a proteasome inhibitor, MG‐132, rescued the WBC100‐induced decline in c‐Myc protein (Figure 4E). To further validate whether WBC100 directly targets c‐Myc protein, we measured c‐Myc mRNA levels in AML MOLM‐13 cells after treatment with WBC100 at various concentrations using RT‐qPCR. We observed that no significant impact on c‐Myc mRNA levels was observed at doses that deplete c‐Myc protein (Figure 4F,G).

We next performed a cycloheximide chase assay and found that WBC100 reduced c‐Myc protein half‐life from 55 to 31 min in MOLM‐13 cells (Figure 4H) and from 50 to 32 min in H9 cells (Figure 4I), respectively, further confirming that WBC100 decreases c‐Myc protein stability.

c‐Myc protein stability is regulated by several mechanisms, the most prominent route for c‐Myc degradation in cells is through the ubiquitin‐proteasome system (UPS).^[^ ^46^, ^47^ ^]^ We next determined the effects of WBC100 on the ubiquitination of wild‐type and different mutants of c‐Myc. We found that WBC100‐mediated ubiquitination for c‐Myc was markedly attenuated by some c‐Myc mutations such as R346A and E351A (Figure 4J). It is known that the ubiquitin E3 ligase CHIP interacts with and ubiquitinates c‐Myc, targeting c‐Myc for degradation by the 26S proteasome.^[^ ^38^ ^]^ To determine whether CHIP was required for WBC100‐mediated c‐Myc ubiquitination in cancer cells, MOLM‐13 cells were treated with WBC100 for 4 h. c‐Myc protein complex was immunoprecipitated using c‐Myc antibody, and then CHIP, Hsp70 were examined by western blotting. We found that WBC100 significantly enhanced the association of CHIP and Hsp70 with c‐Myc (Figure 4K). Moreover, mutants R346A, A321del, and E351A, which are critical for binding of WBC100, decreased WBC100‐dependent binding of CHIP to c‐Myc protein as compared with wild‐type c‐Myc (Figure 4L).To further investigate whether CHIP is required for WBC100‐induced c‐Myc degradation, we performed knockdown of CHIP in Mia‐paca2 cells using siRNA and observed that CHIP‐KD greatly attenuated WBC100‐induced c‐Myc reduction, suggesting that CHIP is critical for WBC100‐mediated c‐Myc degradation (Figure 4M). Because we have demonstrated that CHIP was co‐immunoprecipitated by WBC100 (Figure S2, Supporting Information), we next performed a pull‐down assay using purified recombinant CHIP protein and found that the purified CHIP protein was pulled down by WBC100 (Figure 4N), supporting the direct binding of WBC100 to CHIP protein. All above data indicate that WBC100 might be a molecule glue that selectively induces proteasome‐dependent degradation of c‐Myc protein by interacting with both c‐Myc and E3 ubiquitin ligase CHIP.

### 2.5 WBC100 Potently Regresses c‐Myc Overexpressing Refractory Acute Myeloid Leukemia

To assess in vivo the effect of WBC100 on c‐Myc overexpressing cancer cells of hematological malignancies, we next established an orthotopic human AML model using NOD/SCID/IL2Rγ‐/‐ (NSG) mice with refractory MOLM‐13‐luciferase cells with high c‐Myc levels. Consistent with the in vitro results, orally administered WBC100 twice a day for 20 d exerted dose‐dependent antitumor activity in human c‐Myc overexpressing AML in NSG mice (**Figure** **5**A). Treatment with the higher (0.4 mg kg^–1^ body weight) or medium (0.2 mg kg^–1^ body weight) doses of WBC100 eradicated MOLM‐13‐luciferase cells in vivo (Figure 5A) and all the mice were disease‐free survival on day 35 (Figure 5B). At a low dose (0.1 mg kg^–1^), WBC100 also significantly inhibited tumor growth and prolonged survival of leukemia mice (Figure 5A,B). To determine whether WBC100‐mediated antitumor activity is superior to known c‐Myc transcription inhibitors and chemotherapeutic agents, we compared the antitumor activities of WBC100 with the c‐Myc transcription inhibitor (+)‐JQ1 (BET inhibitor)^[^ ^48^ ^]^ and standard‐of‐care chemotherapeutic agent idarubicin (IDA) against human refractory AML using orthotopic NSG mouse model with MOLM‐13‐luciferase cells. We observed that treatment with 0.8 mg kg^–1^ or 0.4 mg kg^–1^ doses of WBC100 once a day for 14 d eliminated refractory MOLM‐13‐luciferase cells in vivo, but both (+)‐JQ1 (50 mg kg^–1^, intra peritoneal (i.p.), once a day for 14 d) and IDA (1.0 mg kg^–1^, intra venous (i.v.), once a day for 3 d) were ineffective in suppressing tumor growth (Figure S7A,B, Supporting Information). These results indicate that WBC100 exhibits stronger antitumor activity than c‐Myc transcription inhibitor (+)‐JQ1 and standard‐of‐care agent IDA.

Figure 5WBC100 potently regresses c‐Myc overexpressing AML in vivo. A) Bioluminescent images of mice bearing MOLM‐13 leukemia cells (*n* = 5 per group). B) A Kaplan‐Meier survival analysis. C) Gross appearances of tumors from mice of each treatment group. D) Tumor weight of MOLM‐13 xenografts in NSG mice (*n* = 3 per group) treated with WBC100 (0.1 mg, 0.2 mg, and 0.4 mg kg^–1^ orally twice daily) or vehicle. E) Western blot analysis of c‐Myc protein in xenograft tumors from indicated treatment groups with WBC100. F) Representative images of H&E, Ki‐67, c‐Myc, and TUNEL staining of xenograft from each treatment group. Scale bars: 25 µm (H&E, Ki‐67, c‐Myc), 100 µm (TUNEL staining). Green: cell apoptosis staining. Blue: DAPI staining. G) CD33 levels in MOLM‐13, AML‐PDX, and OCI‐Ly3 cells, assessed by western blot. H) Flow cytometry plots showing the gating strategy to determine positive cells. I) CD33, MPO, and CD13 expression in AML‐PDX cells, assessed by Flow cytometry. J) Average tumor volumes of xenografts in AML‐PDX models (*n* = 5 per group) treated with vehicle or WBC100 at indicated time points. K) Average weight and L) gross appearances of tumors in AML PDX models (*n* = 5 per group) after treatment with vehicle or WBC100 for 21 d. M) Representative western blot analysis of c‐Myc protein in PDX tumors from indicated treatment groups with WBC100. All error bars represent means ± s.e.m. Statistical significance was determined by a two‐tailed *t*‐test. **p < 0.01. ****p < 0.0001.

**Figure:**
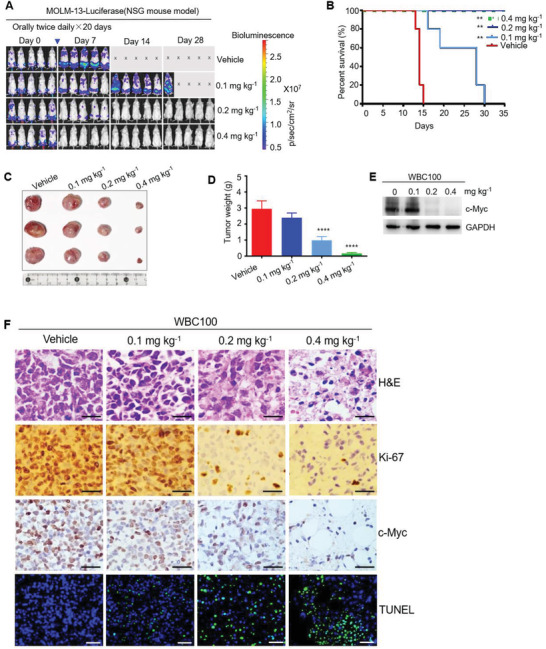

**Figure:**
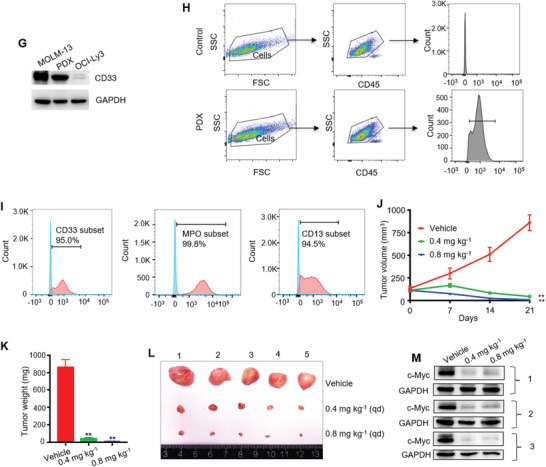

To evaluate the causal correlation between WBC100‐mediated cancer‐killing activity and c‐Myc levels in vivo, we established a subcutaneous xenograft mouse model in NSG mice with MOLM‐13 cells. After xenograft tumors reached 500–800 mm^3^, the mice were randomly divided into four groups to receive either vehicle or various doses of WBC100 (0.1, 0.2, and 0.4 mg kg^–1^) via oral administration twice a day for 7 d and then euthanized. Tumor tissues were processed for H&E staining, immunostaining of Ki‐67 and c‐Myc, and TUNEL staining for apoptosis, and western blot for c‐Myc protein. The vast majority of tumor cells in the WBC100‐treated animals exhibited necrosis, reduced proliferative activity, and increased apoptosis. By contrast, tumors from vehicle‐treated mice displayed histological features of AML with high proliferative activity. Moreover, dose‐dependent decreases of tumor weight, Ki‐67and c‐Myc were observed in WBC100‐treated mice (Figure 5C,D,F). Consistent with these observations, dose‐dependent increased apoptotic cells (green) were also observed (Figure 5F). Western blot analysis revealed positive correlations among tumor reduction, and c‐Myc protein decreases (Figure 5E). These observations indicate that WBC100 kills cancer cells in tumor tissues via targeting c‐Myc in vivo.

To confirm whether WBC100 kills primary c‐Myc overexpressing leukemia cells in vivo, we established a patient‐derived xenograft (PDX) mouse model. Myeloid lineage of PDX was confirmed by western blot analysis of CD33 expression (Figure 5G) and flow cytometric analysis of CD33, CD13, and MPO (markers of acute myeloid lineage cells) (Figure 5H,I). There were 95%, 94.5%, and 99.8% of cells expressing CD33, CD13, and MPO, respectively (Figure 5I). Primary AML cells were directly implanted into flanks of NSG mice (*n* = 5). After xenograft tumors reached ≈100 mm^3^, the mice were randomly divided into three groups to receive either vehicle or WBC100 (0.4 or 0.8 mg kg^–1^) via oral administration once a day for 20 d and then euthanized. Similarly, WBC100 also potently regressed tumors in dose‐dependence in PDX mouse models (Figure 5J–L). Consistently, dose‐dependent decreases of c‐Myc were observed in WBC100‐treated PDX mice (Figure 5M).

### 2.6 WBC100 Potently Regresses c‐Myc Overexpressing Solid Tumors In Vivo

**Figure 6:**
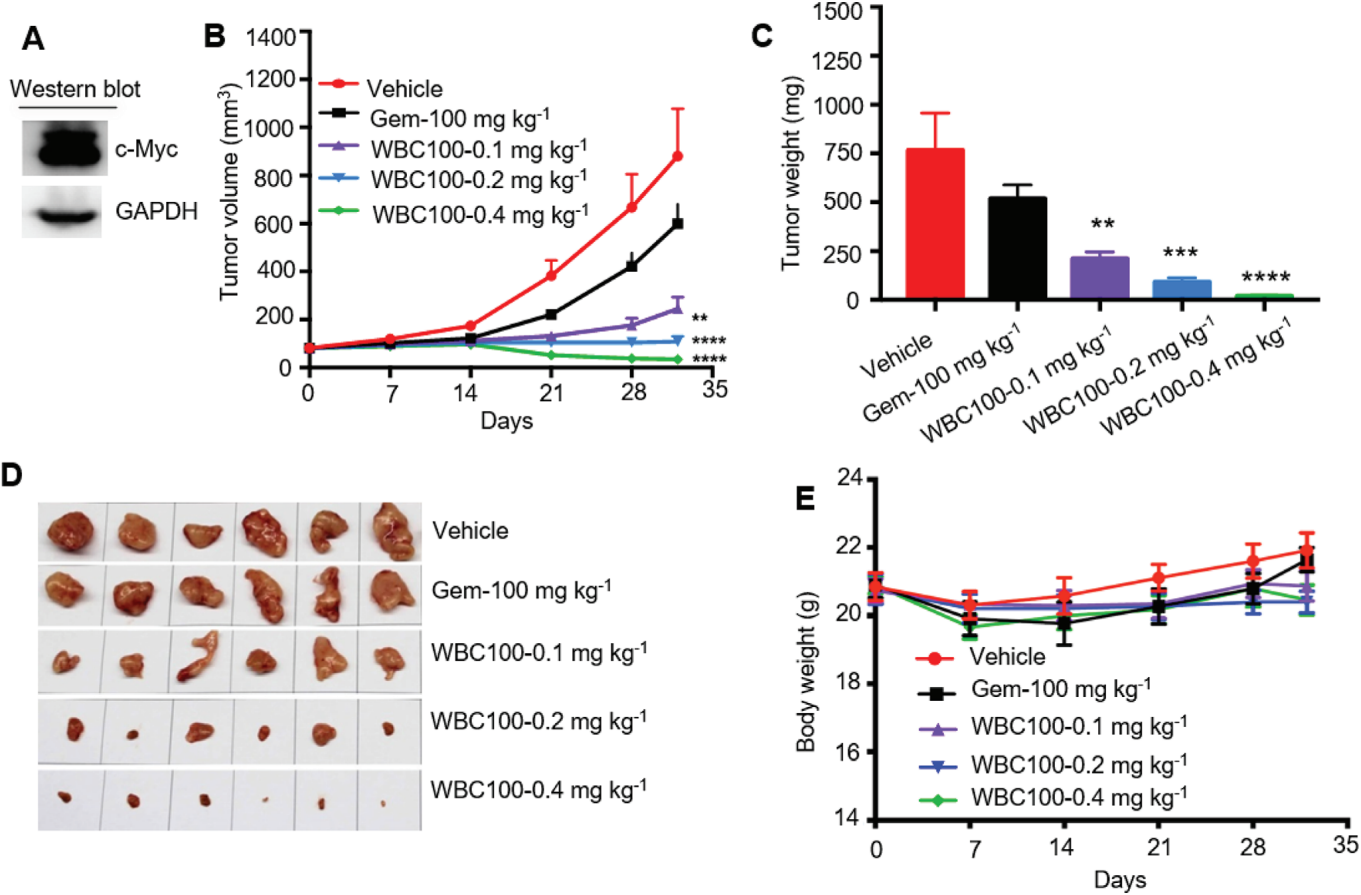
WBC100 potently regresses c‐Myc overexpressing human pancreatic ductal adenocarcinoma (PDAC) in nude mice. A) Western blot analysis of c‐Myc protein in Mia‐paca2 cells. B) Average tumor volumes of Mia‐paca2 xenografts in nude mice (*n* = 10 per group) treated with vehicle or WBC100 or gemcitabine at indicated time points. C) Average weight and D) gross appearances of tumors after treatment with vehicle or WBC100 or gemcitabine for 32 d (*n* = 10 per group). E) Mouse body weight comparison between indicated treatment groups at indicated time points (*n* = 10 per group). All error bars represent means ± s.e.m. Statistical significance was determined by a two‐tailed *t*‐test. **p < 0.01, ***p < 0.001, ****p < 0.0001.

Next, we tested the therapeutic effects of WBC100 on c‐Myc overexpressing pancreatic ductal adenocarcinoma (PDAC), which is a devastating malignancy with almost 90% lethality, emphasizing the need for new therapies, in xenograft mouse models using Mia‐paca2 cells with a high level of c‐Myc protein (**Figure** **6**A). Standard‐of‐care agent gemcitabine (Gem) was used as a positive control. WBC100 treatment led to a marked dose‐dependent decrease in tumor volume and weight at the end of the experiment (Figure 6B–D). Treatment with 0.1, 0.2, and 0.4 mg kg^–1^ doses of WBC100 resulted in 71.94%, 87.63%, and 96.14% of tumor growth inhibition (TGI), respectively (Figure 6C), whereas gemcitabine was modestly effective with 31.83%TGI. WBC100 was also well tolerated by the mice with no significant body weight loss observed (Figure 6E).

To further validate the efficacy of WBC100 on c‐Myc overexpressing tumor in vivo, we established human gastric cancer mouse models using MGC‐803 cell line with high c‐Myc protein level (Figure S8A, Supporting Information) and determined WBC100 activity under the same condition. As expected, WBC100 potently inhibited the growth of MGC‐803 tumor in a dose‐dependent manner. WBC100 exhibited 70.62% of TGI at the low dose of 0.2 mg kg^–1^. Treatment of WBC100 at the dose of 0.4 mg kg^–1^ was sufficient to inhibit tumor growth and the TGI was up to 97.74% (Figure S8B–D, Supporting Information), and the body weight of xenograft mice increased as compared with control (Figure S8E, Supporting Information).

### 2.7 WBC100 Is Well Tolerated in Normal Mice at Efficacious Doses

To assess the tolerability of WBC100, mouse toxicity was evaluated by continuous oral administration (twice daily) of WBC100 to healthy mice (*n* = 6) for two weeks and by monitoring for lethality, mouse weight, blood counts, and blood liver enzyme (alanine amino transferase (ALT)). No systemic toxicity was observed at the dose of 0.4 mg kg^–1^, which is highly efficacious against all c‐Myc overexpressing tumors tested in mouse models. Analyses of body weights, leukocyte subsets, and blood liver enzymes ALT did not reveal any substantial differences between the WBC100‐treated group and control group (Figure S9A–E, Supporting Information), suggesting negligible systemic toxicity of the WBC100. Notably, all of the mice dosed with WBC100 even at as high as 0.8 mg kg^–1^ survived well except for a modest reversible increase in blood liver enzymes and a minor weight loss that were back to normal after 1 week. No obvious abnormalities were observed in the liver, small intestine, spleen, and heart at gross and histological levels (Figure S9F, Supporting Information).

## 3 Discussion

Substantial evidence has highlighted the critical role of c‐Myc oncoprotein in driving tumorigenesis in a majority of human cancers, but no drugs are clinically available for the c‐Myc direct targeting yet. Several small‐molecule c‐Myc inhibitors were reported to cause a reduction of c‐Myc protein levels. For example, MYCi361 could enhance c‐Myc threonine 58 phosphorylation, which consequently decreased c‐Myc protein stability.^[^ ^49^ ^]^ However, the underlying mechanisms for many other small‐molecule c‐Myc inhibitors by which they reduce c‐Myc protein levels remain unclear. One of the most likely explanations seems to be that inhibition of c‐Myc causes cell cycle arrest, which may turn off the transcription of c‐Myc, making the effects largely indirect, such as 10058‐F4.^[^ ^50^ ^]^ Yet WBC100 decreases protein levels via directly inducing c‐Myc protein degradation through proteasome without affecting its mRNA levels, making the effects more direct. Our present study demonstrates that WBC100 is a potent, selective, and efficacious molecule glue that directly targets both c‐Myc protein and CHIP, leading to the degradation of c‐Myc protein. Oral administration of WBC100 exhibits potent regression of tumors in multiple refractory hematological and solid tumor mouse models without serious side effects.

Our study has several important implications. First, it presents the first evidence that the NLS1–Basic–NLS2 region of c‐Myc is a druggable target by a small molecule, which turns the “undruggable” target c‐Myc into a druggable target, thus paving the way for the design of c‐Myc small inhibitors that direct pharmacological inhibition of c‐Myc‐positive cancers. Second, our studies show that WBC100 could bind to both c‐Myc and ubiquitin E3 ligase CHIP, thereby accelerating proteasome‐dependent degradation of c‐Myc protein. This dual activity implies that WBC100 is a selective and efficacious c‐Myc proteolysis targeting molecule. Third, WBC100 can penetrate the nucleus and directly target c‐Myc protein for E3 ligase CHIP‐mediated degradation. Finally, oral administration of WBC100 exhibits potent regression of tumors in refractory hematological and solid tumor mouse models without serious side effects.

In contrast to the BET inhibitor (+)‐JQ1, a well‐known c‐Myc transcription inhibitor that is being tested in clinic,^[^ ^48^ ^]^ exhibits no obvious effect against c‐Myc overexpressing AML MOLM‐13 cells, our study demonstrated that single‐agent WBC100 could potently regress c‐Myc overexpressing AML MOLM‐13 cells, while sparing normal tissues at safe doses. AML is a common lethal human leukemia with a 5‐year overall survival (OS) of <40% using chemotherapies, highlighting the need for new targets and drugs. Treatment with WBC100 at 0.2–0.4 mg kg^–1^ doses potently regressed AML‐MOLM‐13 in vivo and all the mice were disease‐free survival.

Of particular note, our data show that WBC100 could also regress c‐Myc overexpressing pancreatic ductal adenocarcinoma (PDAC) and gastric cancer tested. PDAC is one of the most serious problems in human cancer and its 5‐year survival rate is dismal at <5%. c‐Myc is an important driver in PDAC.^[^ ^51^ ^]^ Treatment with 0.1, 0.2, and 0.4 mg kg^–1^ doses of WBC100 resulted in 71.94%, 87.63%, and 96.14% of TGI, respectively, whereas the TGI of standard‐of‐care agent gemcitabine is only 31.83%. In addition, we demonstrate that treatment of WBC100 at the dose of 0.4 mg kg^–1^ was sufficient to inhibit tumor growth of c‐Myc overexpressing gastric cancer, one of the common gastrointestinal (GI) tumors. Markedly, in contrast to the current chemotherapy agents, c‐Myc overexpressing cancer cells, which carry mutant oncogenes such as MOLM‐13 with *FLT3‐ITD* mutant, Mia‐paca2 with *K‐ras* mutant, and H1975 with *EGFR* mutant (T790M) are also sensitive to WBC100. This suggests that WBC100 may be effective in cases refractory to standard‐of‐care agents.

## 4 Conclusion

In summary, our study reveals several unforeseen properties of WBC100, which overcome some important obstacles related to the design of a clinically viable c‐Myc inhibitor, such as directly targeting nuclear c‐Myc and E3 ligase CHIP for proteasome‐dependent degradation as a potent, selective, and highly efficacious molecule glue. Importantly, the identification of the NLS1–Basic–NLS2 domain as a druggable pocket for directly targeting c‐Myc protein opens new perspectives for pharmacologically intervening c‐Myc in human cancers.

## 5 Experimental Section

### Cell Lines and Culture

In this study, a panel of 39 various human tumor cell lines was used. They included 11 leukemia, 9 lymphoma, 3 multiple myeloma, 12 various solid tumor, and 4 normal cell lines. All media was supplemented with 10% FBS and 1% penicillin‐streptomycin. Human hematological malignancies cell lines, MOLM‐13, KG‐1a, Kasumi‐1, HL‐60, NB4, THP‐1, CEM, Jurkat, MOLT‐4, Nalm6, Raji, Namalwa, Jeko‐1, OCI‐Ly3, OCI‐Ly10, U2932, SU‐DHL16, Pfeiffer, H9, RPMI‐8226, U266, and KM3 were cultured in RPMI‐1640 medium. SEM was cultured in IMDM medium. Human solid tumor cell lines (A549, H1975, H82, Mia‐paca2, PANC‐1, ASPC‐1, HepG2, Huh7, MGC‐803) and human normal cell lines (L02, MRC‐5, WI38, and HEK293T) were maintained in DMEM. SW620, SW480, and HT29 were kept in L‐15 medium. All cells were cultured in a humidified atmosphere containing 5% CO_2_ at 37 °C. Cell lines were routinely tested for mycoplasma. Experiments were performed with noncontaminated cells.

### Chemical Reagents and Antibodies

WBC100 powder was provided by Weben Pharmaceuticals (WBP) and its purity was 99.5%. Owing to light sensitivity, WBC100‐FITC was stored in the dark. FITC (HY‐66019), Cycloheximide (HY‐12320), (+)‐JQ‐1 (HY‐13030) were purchased from MCE (MedChemExpress); MG132 (S2619) from Selleck‐chem; Idarubicin from Hanhui Pharmaceuticals; Gemcitabine from ELI LILLY. CHIP siRNAs were generated by Ribobio company; Lipofectamine 3000 from Thermo (L3000001). Anti‐HA magnetic beads (B26201) from Bimake. Antibodies: GAPDH (60004‐1‐Ig), PD‐L1 (66248‐1‐Ig) from Proteintech; c‐Myc (ab32072) from Abcam; Ubiquitin (1646‐1) from EPIT MICS; Rpb1 (sc‐55492) from Santa Cruz; STAT3 (12640S), XPB (8746), PARP (9532), cleaved‐caspase3 (9664), HA (3724) from Cell signaling Technology; Hsp70 (ET1601‐11), CHIP (ET7108), caspase3 (ET1602), CD33 (for WB, EM1709), Ki‐67 (ER1802‐31) from HuaAn Biotechnology. CD13 PE (652820), CD33 APC (652807), MPO FITC (652821), CD45 PerCP (652803) from BD Biosciences.

### Plasmids

The complete coding region of human c‐Myc (NM_002467) was used. Full‐length c‐Myc and five c‐Myc deletion mutants comprising amino acids 1–143, 1–320, 1–328, 329–439, and 355–439 were inserted into pcDNA3.1(‐), using flanking primers containing restricted enzyme sites (BamHI and EcoRI) and HA‐tag. Full‐length c‐Myc was also inserted into pCDH‐CMV‐MCS‐EF1a‐Puro (SBI, CD510B‐1) and pLVX‐EF1*α*‐mCherry‐N1 (Youbio, VT2012) to generate pCDH‐CMV‐c‐Myc‐flag and pLVX‐EF1*α*‐c‐Myc‐mCherry. shRNA sequences against c‐Myc were inserted into Tet‐pLKO‐puro (Addgene, 21915) to generate c‐Myc knockdown plasmids. All plasmids were constructed using HieffClone One Step Cloning Kit (YEASEN, 10911). Point mutations of c‐Myc (L297A, A321del, L333A, R346A, S347A, E351A, V361A, and Q365A) were generated using Hieff Mut Site‐Directed Mutagenesis Kit (YEASEN, 11003ES10). Target sequences for c‐Myc‐shRNA1 (5’‐GCTTCACCAACAGGAACTATG‐3’) and c‐Myc‐shRNA2 (5’‐GGAACTATGACCTCGACTACG‐3’) were selected using Thermo scientific RNAi designer. All constructs were verified by sequencing. All plasmids used are available from the authors.

### Transfection of Plasmids and siRNAs

Transfections of plasmids were performed using Polyjet transfection reagent (SignaGen, SL100688) according to the manufacturer's instructions. Transfections of siRNAs were performed using Lipofectamine 3000 (Thermo, L3000001) according to the manufacturer's instructions. At 48 h after transfection, cells were used for various experiments. CHIP siRNA (target sequence: 5’‐CTGTGAAGGCGCACTTCTT‐3’) and nontargeting siRNA NC # 1 (siN0000001‐1‐5) were obtained from Ribobio.

### Viral Production and Infection

To produce lentiviruses, HEK293T cells in a 10 cm culture dish were transfected with a solution made of DMEM (500 µL, no FBS) together with of 45 µL Polyjet transfection reagent (SignaGen, SL100688), 3 µg psPAX2 (Addgene, 12260; RRID: Addgene_12260), 3 µg pMD2.G (Addgene, 12259; RRID: Addgene_12259), and 9 µg lentiviral target plasmids (pCDH‐CMV‐c‐Myc‐flag, Tet‐pLKO‐sh‐Myc, empty plasmids). At 16 h after transfection, supernatants of HEK293T cells were gently aspirated and replaced with pre‐warmed (37 °C) culture medium. Viral supernatant was harvested at 48 and 72 h post‐transfection, filtered through Amion Ultra filters (Millipore, UFC910024), and added freshly to the cells along with 1 µg mL^–1^ Polybrene (Santa Cruz, sc‐134220). c‐Myc overexpression or knock‐down cell lines were obtained under 1–2 µg mL^–1^ puromycin (Sangon Biotech, A610593) selection.

### Western Blot Analysis

Cells were collected and washed twice with PBS and then lysed on ice for 30 min in protein extraction reagent (Thermo scientific, 78505) containing protease and phosphatase inhibitor (Thermo scientific, 78443). Cell lysates were centrifuged at 13 000×g for 15 min. The supernatant was collected and heated to 98 °C for 5 min. The protein concentrations were assessed using the Pierce BCA Protein Assay Kit (Thermo scientific, 23225). Equal amounts of protein samples were subjected to SDS‐PAGE and then transferred to PVDF membranes (Bio‐Rad), blocked with 5% nonfat milk in TBS‐T buffer, and incubated with indicated primary antibodies overnight at 4 °C. Commercially valid antibodies were used. All the commercial antibodies were verified by the manufacturers according to the Western blots and/or images on their websites. After three washes with TBS‐T buffer, membranes were probed with a horseradish peroxidase‐conjugated secondary antibody (goat antirabbit (HuaAn Biotechnology, HA1001) or goat antimouse (HuaAn Biotechnology, HA1006)) for 1 h at room temperature, and signals were detected by Tano 5200 Chemiluminescent Imaging System and quantified with ImageJ 1.52a software (https://imagej.nih.gov/ij/). All experiments were repeated at least twice.

### Cell Proliferation and Viability Assays by MTT

WBC100 was dissolved in DMSO to a final concentration of 10 mg mL^–1^ and stored at −20 °C. For MTT (3‐(4,5‐ dimethylthiazol‐2‐yl)‐2,5‐diphenyltetrazolium bromide) assay, cells were seeded in a 96‐well plate with 200 µL medium at a density to maintain untreated cells in an exponential phase of growth during the entire experiment. Cells were cultured for 72 h in the presence of various concentrations of WBC100 followed by incubation with 0.5 mg mL^–1^ MTT (Sangon Biotech, A600799) for 4 h at 37 °C. Lysis buffer (10% SDS, 5% isobutanol, 0.012 m HCl) was added and absorbance was measured at 562 nm 16 h later. The percentage of viable cells was calculated and averaged for each well: percent growth = [(OD treated cells − OD blank)]/ [(OD control cells − OD blank)] × 100. The IC50 defined as the drug concentration that induced a 50% viability decrease was calculated using GraphPad Prism version 7.

### Confocal Laser Microscope Imaging

For detection of localization of WBC100 in cells, FITC labeled WBC100 (WBC100‐FITC) was used. HEK293T cells were transfected with the indicated plasmids for 48 h. Cells grew in confocal dishes and were treated with 10 × 10^−6^ m WBC100‐FITC or FITC for 24 h. Then cells were washed with PBS and the nucleus was stained for 10 min at room temperature using 10 µg mL^–1^ of Hochest 33342 (Thermo Scientific, H21492). Cells were then imaged with Zeiss Confocal Laser Scanning Microscope 710 (LSM710, Germany). ZEISS ZEN Microscope software was used for acquisition and analysis.

### Cellular c‐Myc Protein Competition Pull‐Down Assay with WBC100‐FITC

U2932 cell protein lysates were pre‐incubated with unlabeled WBC100 (10 µg mL^–1^) overnight at 4 °C and then incubated with WBC100‐FITC (0.1 µg mL^–1^) overnight at 4 °C. The mixture was added to 25 µL of anti‐FITC antibody‐bound magnetic beads (Biomag, BMFC300) and mixed by rotation at 4 °C overnight. The supernatant was aspirated and the beads were washed 3 times with PBS. The beads were resuspended in 40 µL of 2 × SDS sample buffer, boiled for 5 min, and subjected to SDS‐PAGE and western blot analyses.

### Binding Assay of c‐Myc Mutants with WBC100‐FITC

To map the binding sites of c‐Myc protein, WBC100‐FITC was used. A series of c‐Myc mutant plasmids were constructed. HEK293T cells were seeded onto 10 cm dishes and transfected with 5 µg of these c‐Myc mutant plasmids using Polyjet transfection reagent (SignaGen, SL100688). At 48 h after transfection, cell samples were harvested and washed twice with cold PBS and lysed in NP40 lysis buffer (50 × 10^−3^ m Tris (pH 7.4), 150 × 10^−3^ m NaCl, 1% NP‐40) containing protease and phosphatase inhibitor (Thermo, 78443) at 4 °C for 30 min. After sonication and centrifugation, 1/20 of the cell lysates were saved as input control. The rest were incubated with WBC100‐FITC (1 × 10^−6^ m) or FITC (1 × 10^−6^ m) at 4 °C overnight on a rotator. For the competition binding assay, cellular proteins were pre‐incubated with unlabeled WBC100 (WBC100 dose was 100‐fold more than WBC100‐FITC) at 4 °C overnight, and then incubated with WBC100‐FITC (1 × 10^−6^ m) at 4 °C overnight. The next day, 10 µL anti‐FITC antibody‐bound magnetic beads (Biomag, BMFC300) were added to each sample and further rotated for 6 h at 4 °C. Beads were washed with NP40 lysis buffer (0.1% Tween‐20) 5 times, then eluted with 2 × SDS sample buffer and boiled at 100 ℃ for 10 min. The supernatant was subjected to western blot.

### Surface Plasmon Resonance

Surface Plasmon Resonance (SPR) experiments were performed using a Biacore T200 (GE Healthcare) instrument by MEDICILON (Shanghai) co., Ltd. The proteins were immobilized on a CM5 (GE Healthcare) chip through amine coupling using the amine coupling kit (GE Healthcare) resulting in immobilization 13742.8 RU level. The samples flowed over the surface with 30 µL min^–1^ for 180 s binding time and 180 s dissociation time. The obtained data were analyzed by the Biacore Evaluation Software 3.0 (GE Healthcare).

### Recombinant Human c‐Myc Protein Pull‐Down Assay with WBC100‐FITC

200 ng recombinant c‐Myc protein (Origene, TP760019, purity > 80%) were incubated with 0.34 µg WBC100‐FITC or with the equal mole FITC in 500 µL PBS overnight at 4 ℃ and then incubated with 10 µL anti‐FITC antibody agarose beads at 4 °C overnight. Samples were washed 3 times in 1 mL cold PBST. The beads were resuspended in 30 µL of SDS‐sample buffer and were boiled for 10 min. The proteins were separated by 8% SDS‐PAGE, immobilized on PVDF membrane, and detected by c‐Myc antibody.

### Recombinant Human CHIP Protein Pull‐Down Assay with WBC100‐Biotin

200 ng recombinant CHIP protein (Novoprotein, C115, purity > 95%) was incubated with WBC100‐biotin (5 × 10^−6^ m) or with the equal mole biotin in 500 µL PBS overnight at 4 °C. For the competition binding assay, CHIP protein was pre‐incubated with unlabeled WBC100 (500 × 10^−6^ m) at 4 °C overnight and then incubated with WBC100 (5 × 10^−6^ m) at 4 °C overnight. The next day, 30 µL streptavidin‐conjugated magnetic beads (Thermo, 88817) were added to each sample and further rotated for 6 h at 4 °C. Samples were washed 3 times in 1 mL cold PBST. The beads were resuspended in 30 µL of SDS‐sample buffer and were boiled for 10 min. The proteins were separated by 10% SDS‐PAGE, immobilized on PVDF membrane, and detected by CHIP antibody.

### RT‐qPCR

RNA from MOLM‐13 cells was isolated using the RNA‐Quick Purification Kit(Esunbio, RN001)according to the manufacturer's instructions. Total cDNA was synthesized using the PrimeScript RT Master Mix (Perfect Real Time) (TAKARA, RR036A). TB Green was used as a DNA intercalator (TAKARA, RR420A) to determine the gene copy number of *β*‐*ACTIN* and *MYC* by qPCR using an ABI 7500 Fast Real‐Time PCR System. 20 µL reaction volume was used containing 2 × TB Green Premix Ex Taq II, 0.8 µL forward primer (10 × 10^−6^ m), 0.8 µL reverse primer (10 × 10^−6^ m), 0.4 µL ROX Reference Dye II, 2 µL cDNA, and 6 µL ddH_2_O. Cycling conditions were 95 °C (30 s), followed by 40 PCR cycles at 95 °C (5 s) and 60 °C (34 s). PCR products were sequenced to ensure the specificity of the primers. Delta‐delta, Ct normalization used *β‐ACTIN* as the reference gene. Data are presented as fold change of relative quantification calculated as 2^−ΔΔCt^, with ∆∆Ct = ∆Ct_treated_ − ∆Ct_control_. Oligonucleotides used for RT‐qPCR: *MYC* (fwd: 5’‐GTCAAGAGGCGAACACACAAC‐3’, rev: 5’‐TTGGACGGACAGGATGTATGC‐3’), *β‐ACTIN* (fwd: 5’‐ACTCTTCCAGCCTTCCTTCC‐3’, rev: 5’‐AGCACTGTGTTGGCGTACAG‐3’). All experiments were repeated at least twice.

### Binding Model of WBC100 with c‐Myc Protein from Computation

The binding model of WBC100 in complex of c‐Myc protein was studied in silico by using docking methods. The homology model of monomer c‐Myc in range of 289–439 a.a. was built by merging its two homology models, i.e., the model of 289–378 a.a. region by using eIF3c (PDB id 4u1c, heterodimer for Eif3c with 27% sequence identity) as a template on SwissModel server and that of 350–439 a.a. region by using OmoMyc structure (PDB id 5i50, homodimer bound to DNA). DNA molecule was removed from the model. The best binding pockets for WBC100 were predicted by implementing the in‐house developed All‐Around Docking (AAD) method. AAD allows a small molecule to dock on the whole surface of a protein to search for the best docking pocket.

### Cycloheximide (CHX) Chase Assay

MOLM‐13 and H9 cells were pretreated with WBC100 (2 × 10^−6^ m) or DMSO for 6 h, followed by cycloheximide (CHX, 25 × 10^−6^ m) treatment. Cells were harvested at the indicated time points and c‐Myc levels were determined by western blot. c‐Myc levels were measured with the densitometric intensity using ImageJ.

### Co‐immunoprecipitation Assay (Co‐IP)

HEK293T cells were transfected with HA‐tagged wild‐type c‐Myc (WT) or HA‐tagged mutants of c‐Myc (R346A, E351A, A321del). At 48 h after transfection, cells were treated with WBC100 (2 × 10^−6^ m) or DMSO in the presence of MG‐132 (10 × 10^−6^ m) for 4 h. Then, cells were lysed with NP40 lysis buffer in the similar way mentioned above. 1/20 of the cell lysates were saved as input control and the rest were incubated with anti‐HA magnetic beads (Bimake, B26201) at 4 °C overnight on a rotator. For endogenous Co‐IP, MOLM‐13 cells were treated with WBC100 (2 × 10^−6^ m) or DMSO in the presence of MG‐132 (10 × 10^−6^ m) for 4 h. Cell lysates were incubated with c‐Myc antibody (Beyotime Biotechnology, AM926) at 4 °C overnight on a rotator. The next day, protein G magnetic beads (Bio‐Rad, 1614023) were added to each sample and further rotated for 12 h at 4 °C. Beads were washed with NP40 lysis buffer (0.1% Tween‐20, 10 × 10^−6^ m MG‐132) for 5 times, then eluted with 2 × SDS sample buffer and boiled at 100 ℃ for 10 min. The supernatant was subjected to western blot.

### Orthotopic Model for AML MOLM‐13 and Xenogen Imaging

To establish an orthotopic model, human AML MOLM‐13 cells were stably transfected with lentiviral firefly luciferase. Cells (1 × 10^6^) were injected through the tail vein into 7‐week‐old female NSG mice (BIOCYTOGEN, B‐CM‐002). After detecting obvious tumor signals (day 7 after cells injection), the mice were randomly divided into four groups to receive either vehicle (sterilized deionized water) or various doses of WBC100 (0.1, 0.2, 0.4 mg kg^–1^) via oral administration twice a day for 20 consecutive days. Bioluminescent imaging of mice was performed at different time points using an in vivo IVIS 100 bioluminescence/optical imaging system (Xenogen, Alameda, CA). Briefly, mice were intraperitoneally injected with d‐Luciferin (2.0 mg per mouse) (Cayman Chemical, 14681) dissolved in PBS 10 min before measuring the luminescence signal. The luminescent imaging was performed once every week.

### AML Subcutaneous Xenograft Model in NSG Mice

MOLM13 (1 × 10^7^) were inoculated subcutaneously in the flank of 7‐week‐old female NSG mice. On day 8 after transplantation, the tumor width and length were measured by one experienced animal technician. Tumor volume was calculated using the formula: (length × width^2^)/2. When the xenograft tumors reached 500–800 mm^3^, the mice were randomly divided into four groups to receive either vehicle (sterilized deionized water) or various doses of WBC100 via oral administration twice a day for 21 d and then euthanized for analyses of tumor weight, H&E staining, western blot and immunohistochemistry (IHC).

### TUNEL Assay

The terminal deoxynucleotidyl transferase‐mediated dUTP nick end labeling (TUNEL) staining was carried out on xenograft sections using the DeadEnd Fluorometric TUNEL System (G3250, Promega), according to the manufacturer's instructions. Briefly, slides containing paraffin‐embedded tissues were de‐waxed in xylene and graded ethanol series then digested with 10 mg mL^–1^ Proteinase K solution for 15–30 min. After washing twice in PBS, the slides were further incubated with TUNEL reaction mixture at 37 ℃ for 1 h. After washing in PBS twice, slides were incubated with DAPI for 10 min and imaged with a Leica DM IL microscope.

### Mouse Tumor Histopathology and IHC Staining

Tumor specimens were prepared and analyzed by board‐certified pathologists. Tumors were fixed in 4% PBS‐buffered formalin; dehydrated and embedded in paraffin, and sectioned and processed for hematoxylin and eosin (H&E) staining (HuaAn Biotechnology). The sections were deparaffinized; heated for antigen retrieval, and incubated with the primary antibodies Ki‐67 and c‐Myc. The secondary antibody was biotinylated with polyclonal antirabbit immunoglobulin (Dako) in combination with streptavidin/horseradish peroxidase. The sections were lightly counterstained using hematoxylin. The slides were visualized on a Zeiss confocal microscope.

### Patient‐Derived Xenograft Experiments

A patient‐derived xenograft (PDX) mouse model was established using primary AML cells from a leukemia patient. Before transplantation, the patient's blood was collected and then peripheral blood mononuclear cells (PBMC) were separated by density gradient centrifugation using Ficoll reagent (TBD Science, LTS1077). Then, PBMC were washed with PBS and lysed with erythrocyte lysis buffer. PBMC (1 × 10^7^ cells) were washed with PBS again and resuspended in 100 µL cold PBS. PDX transplants were generated by mixing 100 µL cells with 100 µL Matrigel and implanted into flanks of NSG mice. After xenograft tumors reached 50mm^3^, the mice were randomly divided into two groups to receive either vehicle (sterilized deionized water) or WBC100 (0.4 mg kg^–1^, 0.8 mg kg^–1^) via oral administration once a day for 20 d and then euthanized.

### Flow Cytometry

For analysis of surface markers, cells were stained in PBS containing 2% BSA. The following fluorescent‐labeled antibodies (purchased from BD Biosciences) were used: CD13 PE (652820), CD33 APC (652807), MPO FITC (652821), and CD45 PerCP (652803). Flow cytometry data were collected using Canto‐I (BD Biosciences) cytometers and analyzed with FlowJo V10 software.

### PDAC Tumor Xenograft Model in Nude Mice

Briefly, 1 × 10^7^ tumor cells of Mia‐Paca2 were inoculated subcutaneously in the flank of 5‐week‐old female BALB/c mice (GemPharmatech, D000521). After the xenograft tumors reached ≈100 mm^3^, the mice were randomly divided into five groups to receive either vehicle (sterilized deionized water) or various doses of WBC100(0.1, 0.2, 0.4 mg kg^–1^) via oral administration twice a day for 28 consecutive days. The positive drug gemcitabine was injected intraperitoneally with a dose of 100 mg kg^–1^ twice a week (BIW) for 4 weeks. Tumor volume and mouse body weight were measured at different time points. At the end of the experiment, all mice were euthanized for analysis of body weight, tumor weight, and survival rate. TGI % was calculated using the following equation: (1 − tumor volume of treated day *x*/tumor volume of control day *x*) × 100.

### Ethics Statement

Primary cell samples were isolated from AML patients and healthy donors with informed consent following the Declaration of Helsinki, respectively. The protocol used was approved by the ethics committee of Second Affiliated Hospital, School of Medicine, Zhejiang University with the approval number of 2018–212. All animal experiments were approved by the ethics committee of Second Affiliated Hospital, School of Medicine, Zhejiang University with the approval number of 2019–048.

### Statistical Analysis

All in vitro data were expressed as mean ± s.d. Animal experiments’ data were expressed as mean ± s.e.m. Statistical analysis (two‐tailed *t*‐test, Pearson correlation, log‐rank test, and Tukey's multiple comparison test) were performed using Prism 7 (GraphPad Software). Sample size (*n*) for each statistical analysis was depicted in each figure legend. Differences with *p*‐value < 0.05 were considered statistically significant. Differences are labeled as follows: *p < 0.05; **p < 0.01; ***p < 0.001; ****p < 0.0001. No sample size calculation was performed. Sample sizes were selected based on animal availability and experience with variation within the immune system. No samples or animals were excluded from the analysis. Animals were randomly assigned to groups. Studies were not conducted blinded.

## Conflict of Interest

The authors declare no conflict of interest.

## Author Contributions

Y.X., Q.F.Y., P.W., Z.X.W., L.Z., S.G.W., and M.Y.L. contributed equally to this work. R.Z.X. and X.X.G. conceived of the study, initiated, designed, and supervised the experiments. R.Z.X., X.X.G., and Y.X. wrote the manuscript. Y.X., Q.F.Y., P.W., Z.X.W., L.Z., S.G.W., M.Y.L., B.W.W., H.F.Z., X.Z.Z., and Y.H. performed experiments. H.L. performed Docking Modeling. H.L.’s work was partially supported by the National Cancer Institute of the National Institutes of Health under award number P30CA033572.

## Supporting information

Supporting Information

## Data Availability

The data that support the findings of this study are available from the corresponding author upon reasonable request.

## References

[1] M. Uhlen , C. Zhang , S. Lee , E. Sjöstedt , L. Fagerberg , G. Bidkhori , R. Benfeitas , M. Arif , Z. Liu , F. Edfors , K. Sanli , K. von Feilitzen , P. Oksvold , E. Lundberg , S. Hober , P. Nilsson , J. Mattsson , J. M. Schwenk , H. Brunnström , B. Glimelius , T. Sjöblom , P. H. Edqvist , D. Djureinovic , P. Micke , C. Lindskog , A. Mardinoglu , F. Ponten , Science 2017, 357, 2507. 10.1126/science.aan2507 28818916

[2] C. Bahr , L. von Paleske , V. V. Uslu , S. Remeseiro , N. Takayama , S. W. Ng , A. Murison , K. Langenfeld , M. Petretich , R. Scognamiglio , P. Zeisberger , A. S. Benk , I. Amit , P. W. Zandstra , M. Lupien , J. E. Dick , A. Trumpp , F. Spitz , Nature 2018, 553, 515. 29342133 10.1038/nature25193

[3] S. A. Abraham , L. E. Hopcroft , E. Carrick , M. E. Drotar , K. Dunn , A. J. Williamson , K. Korfi , P. Baquero , L. E. Park , M. T. Scott , F. Pellicano , A. Pierce , M. Copland , C. Nourse , S. M. Grimmond , D. Vetrie , A. D. Whetton , T. L. Holyoake , Nature 2016, 534, 341. 27281222 10.1038/nature18288 PMC4913876

[4] A. Reddy , J. Zhang , N. S. Davis , A. B. Moffitt , C. L. Love , A. Waldrop , S. Leppa , A. Pasanen , L. Meriranta , M. L. Karjalainen‐Lindsberg , P. Nørgaard , M. Pedersen , A. O. Gang , E. Høgdall , T. B. Heavican , W. Lone , J. Iqbal , Q. Qin , G. Li , S. Y. Kim , J. Healy , K. L. Richards , Y. Fedoriw , L. Bernal‐Mizrachi , J. L. Koff , A. D. Staton , C. R. Flowers , O. Paltiel , N. Goldschmidt , M. Calaminici , et al., Cell 2017, 171, 481.28985567

[5] Y. Gu , J. Zhang , X. Ma , B. W. Kim , H. Wang , J. Li , Y. Pan , Y. Xu , L. Ding , L. Yang , C. Guo , X. Wu , J. Wu , K. Wu , X. Gan , G. Li , L. Li , S. J. Forman , W. C. Chan , R. Xu , W. Huang , Cancer Cell 2017, 32, 115. 28697340 10.1016/j.ccell.2017.06.001 PMC5552197

[6] K. Nakamura , S. Kassem , A. Cleynen , M. L. Chrétien , C. Guillerey , E. M. Putz , T. Bald , I. Förster , S. Vuckovic , G. R. Hill , S. L. Masters , M. Chesi , P. L. Bergsagel , H. Avet‐Loiseau , L. Martinet , M. J. Smyth , Cancer Cell 2018, 33, 634. 29551594 10.1016/j.ccell.2018.02.007

[7] A. S. Farrell , M. M. Joly , B. L. Allen‐Petersen , P. J. Worth , C. Lanciault , D. Sauer , J. Link , C. Pelz , L. M. Heiser , J. P. Morton , N. Muthalagu , M. T. Hoffman , S. L. Manning , E. D. Pratt , N. D. Kendsersky , N. Egbukichi , T. S. Amery , M. C. Thoma , Z. P. Jenny , A. D. Rhim , D. J. Murphy , O. J. Sansom , H. C. Crawford , B. C. Sheppard , R. C. Sears , Nat. Commun. 2017, 8, 1728. 29170413 10.1038/s41467-017-01967-6 PMC5701042

[8] S. Mueller , T. Engleitner , R. Maresch , M. Zukowska , S. Lange , T. Kaltenbacher , B. Konukiewitz , R. Öllinger , M. Zwiebel , A. Strong , H. Y. Yen , R. Banerjee , S. Louzada , B. Fu , B. Seidler , J. Götzfried , K. Schuck , Z. Hassan , A. Arbeiter , N. Schönhuber , S. Klein , C. Veltkamp , M. Friedrich , L. Rad , M. Barenboim , C. Ziegenhain , J. Hess , O. M. Dovey , S. Eser , S. Parekh , et al., Nature 2018, 554, 62. 29364867 10.1038/nature25459 PMC6097607

[9] X. Wang , Z. Huang , Q. Wu , B. C. Prager , S. C. Mack , K. Yang , L. J. Y. Kim , R. C. Gimple , Y. Shi , S. Lai , Q. Xie , T. E. Miller , C. G. Hubert , A. Song , Z. Dong , W. Zhou , X. Fang , Z. Zhu , V. Mahadev , S. Bao , J. N. Rich , Cancer Res. 2017, 77, 4947. 28729418 10.1158/0008-5472.CAN-17-0114 PMC5600855

[10] S. Bolin , A. Borgenvik , C. U. Persson , A. Sundström , J. Qi , J. E. Bradner , W. A. Weiss , Y. J. Cho , H. Weishaupt , F. J. Swartling , Oncogene 2018, 37, 2850. 29511348 10.1038/s41388-018-0135-1 PMC5966365

[11] M. J. Topper , M. Vaz , K. B. Chiappinelli , C. E. DeStefano Shields , N. Niknafs , R. C. Yen , A. Wenzel , J. Hicks , M. Ballew , M. Stone , P. T. Tran , C. A. Zahnow , M. D. Hellmann , V. Anagnostou , P. L. Strissel , R. Strick , V. E. Velculescu , S. B. Baylin , Cell 2017, 171, 1284. 29195073 10.1016/j.cell.2017.10.022 PMC5808406

[12] A. Bouillez , H. Rajabi , S. Pitroda , C. Jin , M. Alam , A. Kharbanda , A. Tagde , K. K. Wong , D. Kufe , Cancer Res. 2016, 76, 1538. 26833129 10.1158/0008-5472.CAN-15-1804 PMC4794417

[13] C. L. Christensen , N. Kwiatkowski , B. J. Abraham , J. Carretero , F. Al‐Shahrour , T. Zhang , E. Chipumuro , G. S. Herter‐Sprie , E. A. Akbay , A. Altabef , J. Zhang , T. Shimamura , M. Capelletti , J. B. Reibel , J. D. Cavanaugh , P. Gao , Y. Liu , S. R. Michaelsen , H. S. Poulsen , A. R. Aref , D. A. Barbie , J. E. Bradner , R. E. George , N. S. Gray , R. A. Young , K. K. Wong , Cancer Cell 2014, 26, 909. 25490451 10.1016/j.ccell.2014.10.019 PMC4261156

[14] G. Mollaoglu , M. R. Guthrie , S. Böhm , J. Brägelmann , I. Can , P. M. Ballieu , A. Marx , J. George , C. Heinen , M. D. Chalishazar , H. Cheng , A. S. Ireland , K. E. Denning , A. Mukhopadhyay , J. M. Vahrenkamp , K. C. Berrett , T. L. Mosbruger , J. Wang , J. L. Kohan , M. E. Salama , B. L. Witt , M. Peifer , R. K. Thomas , J. Gertz , J. E. Johnson , A. F. Gazdar , R. J. Wechsler‐Reya , M. L. Sos , T. G. Oliver , Cancer Cell 2017, 31, 270. 28089889 10.1016/j.ccell.2016.12.005 PMC5310991

[15] H. Dang , A. Takai , M. Forgues , Y. Pomyen , H. Mou , W. Xue , D. Ray , K. C. H. Ha , Q. D. Morris , T. R. Hughes , X. W. Wang , Cancer Cell 2017, 32, 101. 28697339 10.1016/j.ccell.2017.06.002 PMC5539779

[16] P. Liu , M. Ge , J. Hu , X. Li , L. Che , K. Sun , L. Cheng , Y. Huang , M. G. Pilo , A. Cigliano , G. M. Pes , R. M. Pascale , S. Brozzetti , G. Vidili , A. Porcu , A. Cossu , G. Palmieri , M. C. Sini , S. Ribback , F. Dombrowski , J. Tao , D. F. Calvisi , L. Chen , X. Chen , Hepatology 2017, 66, 167. 28370287 10.1002/hep.29183 PMC5481473

[17] J. Welti , A. Sharp , W. Yuan , D. Dolling , D. Nava Rodrigues , I. Figueiredo , V. Gil , A. Neeb , M. Clarke , G. Seed , M. Crespo , S. Sumanasuriya , J. Ning , E. Knight , J. C. Francis , A. Hughes , W. S. Halsey , A. Paschalis , R. S. Mani , G. V. Raj , S. R. Plymate , S. Carreira , G. Boysen , A. M. Chinnaiyan , A. Swain , J. S. de Bono , Clin. Cancer Res. 2018, 24, 3149. 29555663 10.1158/1078-0432.CCR-17-3571

[18] V. R. Minciacchi , C. Spinelli , M. Reis‐Sobreiro , L. Cavallini , S. You , M. Zandian , X. Li , R. Mishra , P. Chiarugi , R. M. Adam , E. M. Posadas , G. Viglietto , M. R. Freeman , E. Cocucci , N. A. Bhowmick , D. Di Vizio , Cancer Res. 2017, 77, 2306. 28202510 10.1158/0008-5472.CAN-16-2942

[19] E. Chipumuro , E. Marco , C. L. Christensen , N. Kwiatkowski , T. Zhang , C. M. Hatheway , B. J. Abraham , B. Sharma , C. Yeung , A. Altabef , A. Perez‐Atayde , K. K. Wong , G. C. Yuan , N. S. Gray , R. A. Young , R. E. George , Cell 2014, 159, 1126. 25416950 10.1016/j.cell.2014.10.024 PMC4243043

[20] A. R. Wasylishen , L. Z. Penn , Genes Cancer 2010, 1, 532. 21779457 10.1177/1947601910378024 PMC3092215

[21] M. Eilers , R. N. Eisenman , Genes Dev. 2008, 22, 2755. 18923074 10.1101/gad.1712408 PMC2751281

[22] S. Walz , F. Lorenzin , J. Morton , K. E. Wiese , B. von Eyss , S. Herold , L. Rycak , H. Dumay‐Odelot , S. Karim , M. Bartkuhn , F. Roels , T. Wüstefeld , M. Fischer , M. Teichmann , L. Zender , C. L. Wei , O. Sansom , E. Wolf , M. Eilers , Nature 2014, 511, 483. 25043018 10.1038/nature13473 PMC6879323

[23] D. G. McFadden , K. Politi , A. Bhutkar , F. K. Chen , X. Song , M. Pirun , P. M. Santiago , C. Kim‐Kiselak , J. T. Platt , E. Lee , E. Hodges , A. P. Rosebrock , R. T. Bronson , N. D. Socci , G. J. Hannon , T. Jacks , H. Varmus , Proc. Natl. Acad. Sci. USA 2016, 113, E6409. 27702896 10.1073/pnas.1613601113 PMC5081629

[24] D. S. C. Butler , C. Cafaro , J. Putze , M. L. Y. Wan , T. H. Tran , I. Ambite , S. Ahmadi , S. Kjellström , C. Welinder , S. M. Chao , U. Dobrindt , C. Svanborg , Nat. Biotechnol. 2021, 39, 754. 33574609 10.1038/s41587-020-00805-3

[25] M. L. Giardino Torchia , J. D. Ashwell , Proc. Natl. Acad. Sci. USA 2018, 115, 9821. 30201726 10.1073/pnas.1813867115 PMC6176636

[26] W. C. Gustafson , J. G. Meyerowitz , E. A. Nekritz , J. Chen , C. Benes , E. Charron , E. F. Simonds , R. Seeger , K. K. Matthay , N. T. Hertz , M. Eilers , K. M. Shokat , W. A. Weiss , Cancer Cell 2014, 26, 414. 25175806 10.1016/j.ccr.2014.07.015 PMC4160413

[27] A. Wiegering , F. W. Uthe , T. Jamieson , Y. Ruoss , M. Hüttenrauch , M. Küspert , C. Pfann , C. Nixon , S. Herold , S. Walz , L. Taranets , C. T. Germer , A. Rosenwald , O. J. Sansom , M. Eilers , Cancer Discovery 2015, 5, 768. 25934076 10.1158/2159-8290.CD-14-1040 PMC5166973

[28] C. V. Dang , E. P. Reddy , K. M. Shokat , L. Soucek , Nat. Rev. Cancer 2017, 17, 502. 28643779 10.1038/nrc.2017.36 PMC5945194

[29] H. Wang , D. I. Hammoudeh , A. V. Follis , B. E. Reese , J. S. Lazo , S. J. Metallo , E. V. Prochownik , Mol. Cancer Ther. 2007, 6, 2399. 17876039 10.1158/1535-7163.MCT-07-0005

[30] A. V. Follis , D. I. Hammoudeh , H. Wang , E. V. Prochownik , S. J. Metallo , Chem. Biol. 2008, 15, 1149. 19022175 10.1016/j.chembiol.2008.09.011

[31] D. I. Hammoudeh , A. V. Follis , E. V. Prochownik , S. J. Metallo , J. Am. Chem. Soc. 2009, 131, 7390. 19432426 10.1021/ja900616b

[32] J. Guo , R. A. Parise , E. Joseph , M. J. Egorin , J. S. Lazo , E. V. Prochownik , J. L. Eiseman , Cancer Chemother. Pharmacol. 2009, 63, 615. 18509642 10.1007/s00280-008-0774-y PMC2752825

[33] D. M. Clausen , J. Guo , R. A. Parise , J. H. Beumer , M. J. Egorin , J. S. Lazo , E. V. Prochownik , J. L. Eiseman , J. Pharmacol. Exp. Ther. 2010, 335, 715. 20801893 10.1124/jpet.110.170555 PMC2993546

[34] K. Y. Jung , H. Wang , P. Teriete , J. L. Yap , L. Chen , M. E. Lanning , A. Hu , L. J. Lambert , T. Holien , A. Sundan , N. D. Cosford , E. V. Prochownik , S. Fletcher , J. Med. Chem. 2015, 58, 3002. 25734936 10.1021/jm501440q PMC4955407

[35] S. Vispé , L. DeVries , L. Créancier , J. Besse , S. Bréand , D. J. Hobson , J. Q. Svejstrup , J. P. Annereau , D. Cussac , C. Dumontet , N. Guilbaud , J. M. Barret , C. Bailly , Mol. Cancer Ther. 2009, 8, 2780. 19808979 10.1158/1535-7163.MCT-09-0549

[36] N. Beglyarova , E. Banina , Y. Zhou , R. Mukhamadeeva , G. Andrianov , E. Bobrov , E. Lysenko , N. Skobeleva , L. Gabitova , D. Restifo , M. Pressman , I. G. Serebriiskii , J. P. Hoffman , K. Paz , D. Behrens , V. Khazak , S. A. Jablonski , E. A. Golemis , L. M. Weiner , I. Astsaturov , Clin. Cancer Res. 2016, 22, 6153. 27384421 10.1158/1078-0432.CCR-16-0149 PMC5161635

[37] A. Yang , S. Qin , B. A. Schulte , S. P. Ethier , K. D. Tew , G. Y. Wang , Cancer Res. 2017, 77, 6641. 28951456 10.1158/0008-5472.CAN-16-3452 PMC5712265

[38] I. Paul , S. F. Ahmed , A. Bhowmik , S. Deb , M. K. Ghosh , Oncogene 2013, 32, 1284. 22543587 10.1038/onc.2012.144

[39] H. Yang , T. W. Li , Y. Zhou , H. Peng , T. Liu , E. Zandi , M. L. Martínez‐Chantar , J. M. Mato , S. C. Lu , Antioxid. Redox Signaling 2015, 22, 259. 10.1089/ars.2014.6027 PMC4283066 25226451

[40] Z. M. Mu , X. Y. Yin , E. V. Prochownik , J. Biol. Chem. 2002, 277, 43175. 12196529 10.1074/jbc.M206066200

[41] L. A. Jung , A. Gebhardt , W. Koelmel , C. P. Ade , S. Walz , J. Kuper , B. von Eyss , S. Letschert , C. Redel , L. d'Artista , A. Biankin , L. Zender , M. Sauer , E. Wolf , G. Evan , C. Kisker , M. Eilers , Oncogene 2017, 36, 1911. 27748763 10.1038/onc.2016.354

[42] S. Sammak , N. Hamdani , F. Gorrec , M. D. Allen , S. M. V. Freund , M. Bycroft , G. Zinzalla , Biochemistry 2019, 58, 3144. 31260268 10.1021/acs.biochem.9b00296 PMC6791285

[43] S. K. Nair , S. K. Burley , Cell 2003, 112, 193. 12553908 10.1016/s0092-8674(02)01284-9

[44] A. Waterhouse , M. Bertoni , S. Bienert , G. Studer , G. Tauriello , R. Gumienny , F. T. Heer , T. A. P. de Beer , C. Rempfer , L. Bordoli , R. Lepore , T. Schwede , Nucleic Acids Res. 2018, 46, W296. 29788355 10.1093/nar/gky427 PMC6030848

[45] D. D. Yu , S. S. Andrali , H. Li , M. Lin , W. Huang , B. M. Forman , Bioorg. Med. Chem. 2016, 24, 3986. 27372840 10.1016/j.bmc.2016.06.039

[46] L. R. Thomas , W. P. Tansey , Adv. Cancer Res. 2011, 110, 77. 21704229 10.1016/B978-0-12-386469-7.00004-9

[47] X. X. Sun , Y. Chen , Y. Su , X. Wang , K. M. Chauhan , J. Liang , C. J. Daniel , R. C. Sears , M. S. Dai , Proc. Natl. Acad. Sci. USA 2018, 115, 10983. 30305424 10.1073/pnas.1802932115 PMC6205424

[48] J. A. Mertz , A. R. Conery , B. M. Bryant , P. Sandy , S. Balasubramanian , D. A. Mele , L. Bergeron , R. J. Sims 3rd , Proc. Natl. Acad. Sci. USA 2011, 108, 16669. 21949397 10.1073/pnas.1108190108 PMC3189078

[49] H. Han , A. D. Jain , M. I. Truica , J. Izquierdo‐Ferrer , J. F. Anker , B. Lysy , V. Sagar , Y. Luan , Z. R. Chalmers , K. Unno , H. Mok , R. Vatapalli , Y. A. Yoo , Y. Rodriguez , I. Kandela , J. B. Parker , D. Chakravarti , R. K. Mishra , G. E. Schiltz , S. A. Abdulkadir , Cancer Cell 2019, 36, 483. 31679823 10.1016/j.ccell.2019.10.001 PMC6939458

[50] M. J. Huang , Y. C. Cheng , C. R. Liu , S. Lin , H. E. Liu , Exp. Hematol. 2006, 34, 1480. 17046567 10.1016/j.exphem.2006.06.019

[51] M. Wirth , S. Mahboobi , O. H. Krämer , G. Schneider , Mol. Cancer Ther. 2016, 15, 1792. 27406986 10.1158/1535-7163.MCT-16-0050

