# Supplementary material for: A Selective Small‐Molecule c‐Myc Degrader Potently Regresses Lethal c‐Myc Overexpressing Tumors
Source: Adv Sci (Weinh). 2022 Jan 20;9(8):2104344. doi: 10.1002/advs.202104344 (PMC8922104; doi:10.1002/advs.202104344)
Supplement: Supplementary file 1 — Supporting Information [file ADVS-9-2104344-s001.pdf]

ADVANCED SCIENCE
Open Access

Supporting Information

for *Adv. Sci.*, DOI 10.1002/advs.202104344

A Selective Small-Molecule c-Myc Degrader Potently Regresses Lethal c-Myc Overexpressing Tumors

Ying Xu, Qingfeng Yu, Ping Wang, Zhaoxing Wu, Lei Zhang, Shuigao Wu, Mengyuan Li, Bowen Wu, Hongzhi Li, Haifeng Zhuang, Xuzhao Zhang, Yu Huang, Xiaoxian Gan and Rongzhen Xu**

© 2020 Wiley-VCH GmbH

ADVANCED SCIENCE
Open Access

Supporting Information

for *Adv. Sci.,* DOI: 10.1002/advs.202104344

A Selective Small-molecule c-Myc Degrader Potently Regresses Lethal c-Myc Over-expressing Tumors

Ying Xu, Qingfeng Yu, Ping Wang, Zhaoxing Wu, Lei Zhang, Shuigao Wu, Mengyuan Li, Bowen Wu, Hongzhi Li, Haifeng Zhuang, Xuzhao Zhang, Yu Huang, Xiaoxian Gan, and Rongzhen Xu **

Supporting Information

A Selective Small-molecule c-Myc Degrader Potently Regresses Lethal c-Myc Over-expressing Tumors

Ying Xu, Qingfeng Yu, Ping Wang, Zhaoxing Wu, Lei Zhang, Shuigao Wu, Mengyuan Li, Bowen Wu, Hongzhi Li, Haifeng Zhuang, Xuzhao Zhang, Yu Huang, Xiaoxian Gan, and Rongzhen Xu **

Supplementary Experimental methods

Normal primary cells and culture: the healthy donors' blood was collected and then peripheral blood mononuclear cells (PBMC) were separated by density gradient centrifugation using Ficoll reagent (TBD Science, LTS1077). Then, PBMC were washed with PBS and lysed with erythrocyte lysis buffer. PBMC were cultured in IMDM medium supplemented with 20% FBS and 1% penicillin-streptomycin. All cells were cultured in a humidified atmosphere containing 5% CO_2 at 37 °C.

Target Pull-down Experiment: Whole cell lysates were incubated with 5 μM WBC100-biotin or biotin as control. Proteins were then purified using streptavidin-conjugated magnetic beads (Thermo, 88817) for 6 hours at 4 ℃. After washed and eluted, the bound proteins were resolved in SDS-PAGE and then visualized by silver stain using Pierce™ Silver Stain Kit (Thermo, 24612). Then, specific bands were cut off and identified by mass spectrometry.

Detection of Localization of WBC100 in MOLM-13 and A549 cells: MOLM-13 and A549 cells were treated with FITC labeled WBC100 (WBC100-FITC) at 50 nM for 24 hours. MOLM-13 cells were harvested and fixed on slides with 4% paraformaldehyde for 30 min. Then cells were washed with PBS and the nucleus was stained for 20 min at room temperature using 10 μg mL^{-1} of Hochest 33342 (Thermo Scientific, H21492). ZEISS ZEN Microscope software was used for acquisition and analysis.

Detection of WBC100 Content by FCM: For detection of WBC100 content in MOLM13 and A549 cells, FITC labeled WBC100 (WBC100-FITC) was used. Cells were cultured (6-well) in triplicate at 2×10^5 cells/mL and treated with 50 nM WBC100-FITC or DMSO for 24 hours. The cells were collected and washed twice with PBS, suspended in PBS. The absorbing rates for WBC100-FITC were performed on a Canto-Ⅱ (BD Biosciences) by detecting FITC fluorescence.

Detection of Apoptotic Cells by FCM: MOLM-13 cells were treated with WBC100 for 24, 48, and 72 hours at indicated concentration, centrifuged, and washed with PBS. Cells

were incubated with 200 μL of binding buffer containing Annexin V–FITC and PI (Liankebio, 70-AP101-100) for 15 min at 20 °C in the dark. 300 μL of PBS was added before flow cytometry analysis. For each sample, 1×10^4 cells were analyzed by flow cytometry in a DxFLEX flow cytometer (Beckman Coulter). Fluorescence was collected at 525 nm (FITC) and 585 nm (PI). Cells were gated based on FSC-A/SSC-A. Positive gates were set using blank controls and single dye controls simultaneously. Data were analyzed using FlowJo V10. All experiments were repeated at least three times.

Comparison of WBC100-mediated Anti-tumor Activity with (+)-JQ1 and Idarubicin in Orthotopic Model for AML MOLM-13: To establish an orthotopic model, human AML MOLM-13-luciferase cells (1×10^6) were injected through the tail vein into 7-week-old female NSG mice (BIOCYTOGEN, B-CM-002). After detecting obvious tumor signal, the mice were randomly divided into indicated groups to receive either vehicle (sterilized deionized water) or various doses of WBC100 (0.4, 0.8 mg kg^{-1}) or (+)-JQ1 (50 mg kg^{-1}) via oral administration once a day for 14 consecutive days. IDA (1.0 mg kg^{-1}) was administered through the tail vein for 3 days. Bioluminescent imaging of mice was performed at different time points using an in vivo IVIS 100 bioluminescence/optical imaging system (Xenogen, Alameda, CA).

Solid Tumor Xenograft Model in NOD/SCID Mice: Briefly, 1×10^7 MGC-803 cells were inoculated subcutaneously in the flank of 5-week-old female NOD/SCID mice (SLAC Laboratory Animal Co., Ltd). After the xenograft tumors reached 200-300 mm^3, the mice were randomly divided into three groups to receive either vehicle (sterilized deionized water) or various doses of WBC100 (0.2, 0.4 mg kg^{-1}) via oral administration twice a day for 20 consecutive days. Tumor volume and mouse body weight were measured at different time points. At the end of the experiments, all mice were euthanized for analysis of body weight, tumor weight.

Toxicity Evaluation of WBC100 on Healthy Mice: In this study, ICR（Institute of Cancer Research）mice were used to evaluate the toxicity of WBC100. Briefly, WBC100 was administered twice daily via oral gavage to mice for 14 consecutive days. Male and female 7-week-old mice (n = 6, 3 female and 3 males, 20 g) were assigned to groups. Assessment of toxicity was based on mortality, clinical observations, body weight, food consumption, clinical and anatomic pathology, blood counts (WBC, PLT, and HGB), and blood liver enzyme alanine amino transferase (ALT) at the indicated time points. At the end of observation, mice were killed and organs (small intestine, liver, heart, and spleen) were

fixed in 10% neutral buffered formalin for 48 hours at 4 °C and transferred to 70% ethanol before paraffin processing at Zhejiang Chinese Medical University pathological center. Paraffin sections were deparaffinized and rehydrated, stained with hematoxylin and eosin.

Synthesis and Characterization of WBC100:

General method: All reagents and solvents were reagent grade and used without further purification. The reactions were monitored using HPLC instruments.

HPLC: Agilent Technologies 1260, UV detection was done at 220 nm, Agilent Infinity Poroshell Lab EC-C18, 4.6 mm×150 mm, column at 30 °C, at a flow rate of 1 mL min^{-1} using water (Containing 0.1V/V% Trifluoroacetic acid) and MeCN as eluents.

1H NMR and proton-decoupled 13C NMR measurements were performed on Zhongke-Niujin WNMR-I- 400 Superconducting NMR Spectrometer (400 MHz for 1H NMR, 100 MHz for 13C NMR), using DMSO-d6 as solvent. 1H and 13C NMR data are in the form of δ values, given in part per million (ppm).

HRMS were measured on an Agilent 6224 TOF LC/MS instrument and Elemental analyses were recorded using a VarioMICRO Elemental Analyzer.

14-dextrorotary-valyl-triptolide (14-D-Valine-Triptolide, WBC100)

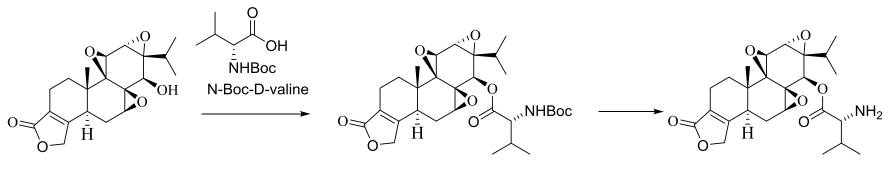

Step A: *N*-Boc-D-valine (700 mg, 3.22 mmol, 2.3 eq.) and 4-dimethylaminopyridine (424 mg, 3.47 mmol, 2.5 eq.) were dissolved in dichloromethane (10 mL), and cooled to 0 °C. 1-Ethyl-3-(3-dimethylaminopropyl) carbodiimide hydrochloride (610 mg, 3.18 mmol, 2.3 eq.) and Triptolide (TPL) (500 mg, 1.39 mmol, 1.0 eq.) were added into the mixed solution at 0 °C and reacted for 4 hours at 5 °C. The reaction solution was diluted with dichloromethane and washed with 5% sodium bicarbonate solution, and then washed with 0.1 M aqueous HCl solution, the organic layer dried over Na_2SO_4, filtered and the filtrate was concentrated under reduced pressure to obtain 776 mg 14-*N*-Boc-D -Valine-TPL as a white solid.

Step B: The product 14-*N*-Boc-D -Valine-TPL (776 mg) in the previous step was dissolved in dichloromethane (10 mL) and cooled to 0 °C, then 5 mL of trifluoroacetic acid was added dropwise. After the addition, the reaction solution was reacted at 10 °C for 6 hours. The reaction solution was diluted with dichloromethane and carefully neutralized with 5% sodium bicarbonate solution, the organic phase was washed with brine, and then dried over

Na2SO4, filtered and the filtrate was concentrated under reduced pressure to give a crude product. The crude product was recrystallized with isopropyl acetate to obtain 500 mg 14-D-Valine-Triptolide (WBC100) as a white solid (1.09 mmol, 78% for 2 steps).

1H NMR (400 MHz, DMSO-d_6, δ): 4.98 (s, 1H), 4.89 - 4.75 (m, 2H), 3.94 (d, *J* = 3.2 Hz, 1H), 3.69 (d, *J* = 2.8 Hz, 1H), 3.58 (d, *J* = 5.6 Hz, 1H), 3.21 (d, *J* = 4.8 Hz, 1H), 2.65 -2.61 (m, 1H), 2.26 -2.19 (m, 1H), 2.14 -2.09 (m, 1H), 2.02 - 1.96 (m, 2H), 1.87 -1.78 (m, 2H), 1.66 (s, 2H), 1.32 -1.27 (m, 2H), 0.94 (d, *J* = 6.8 Hz, 3H), 0.91 - 0.89 (m, 9H), 0.76 (d, *J* = 7.2 Hz, 3H). 13C NMR (100 MHz, DMSO-d_6, δ): 174.64, 173.11, 162.26, 123.11, 71.25, 70.21, 63.45, 62.66, 60.38, 59.52, 59.20, 54.69, 54.50, 39.82, 35.06, 31.86, 28.93, 27.42, 22.36, 18.90, 17.52, 17.06, 16.57, 16.49, 13.51. HRMS calculated for C25H33NO7: 459.5390; found 460.2334 $[M+H]^+$. Elementary analysis (% calcd, % found for C25H33NO7): C, (65.34, 65.39); H, (7.24, 7.31); N, (3.05, 2.93).

1H NMR 400 MHz DMSO-d_6

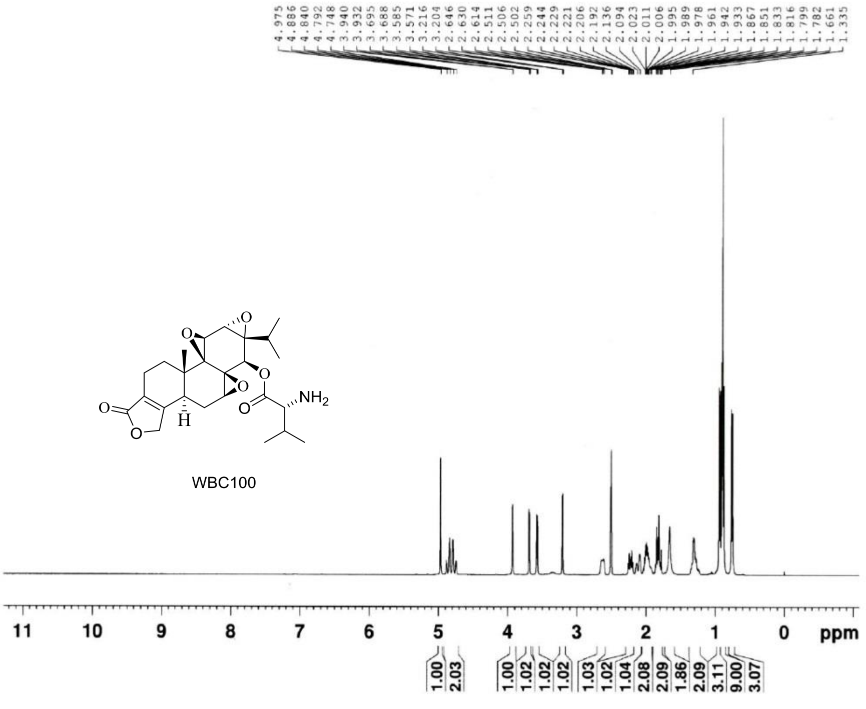

13C NMR 100 MHz DMSO-d_6

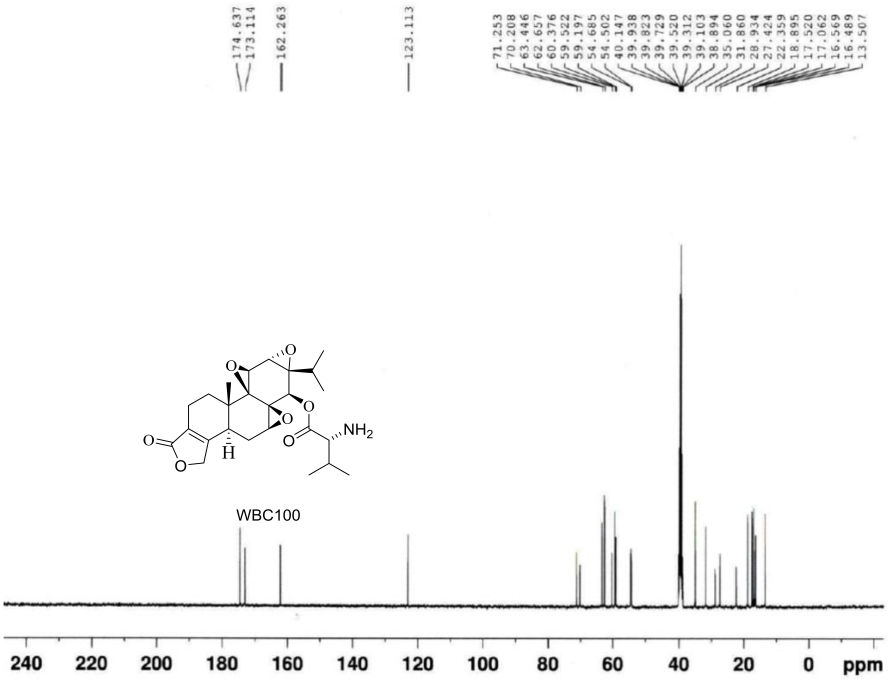

13C NMR 100 MHz DMSO-d_6 (DEPT 135◦)

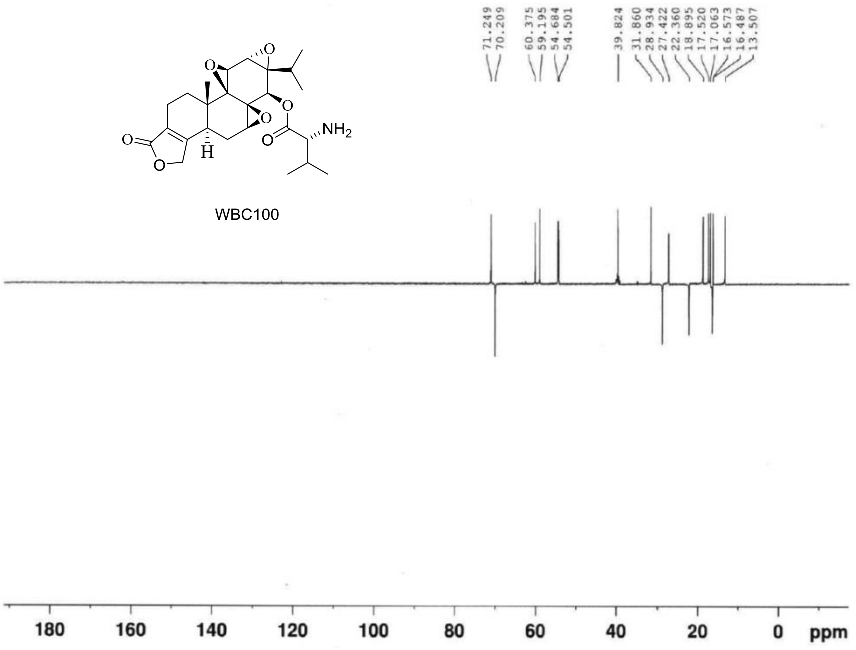

Supplementary Figures

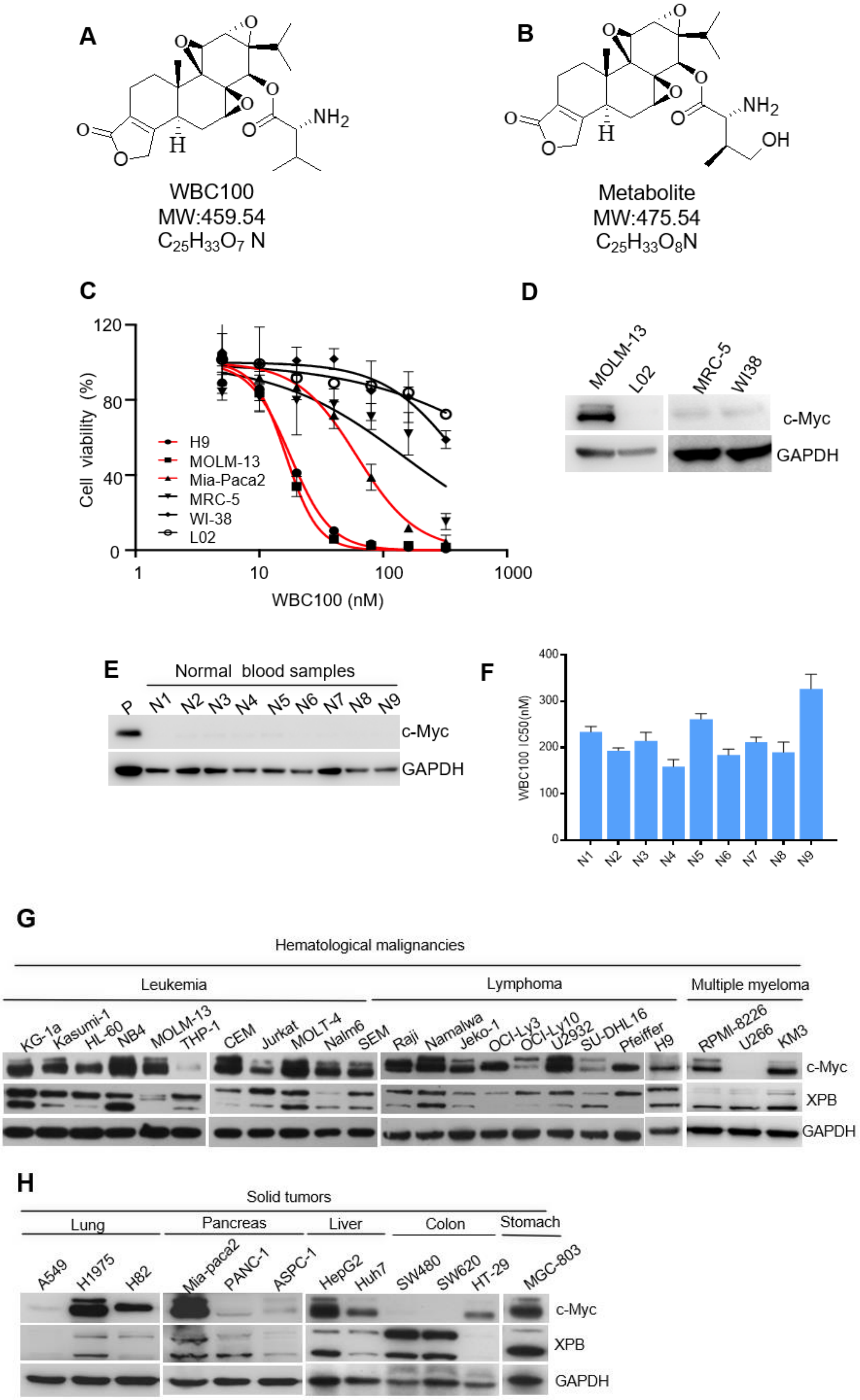

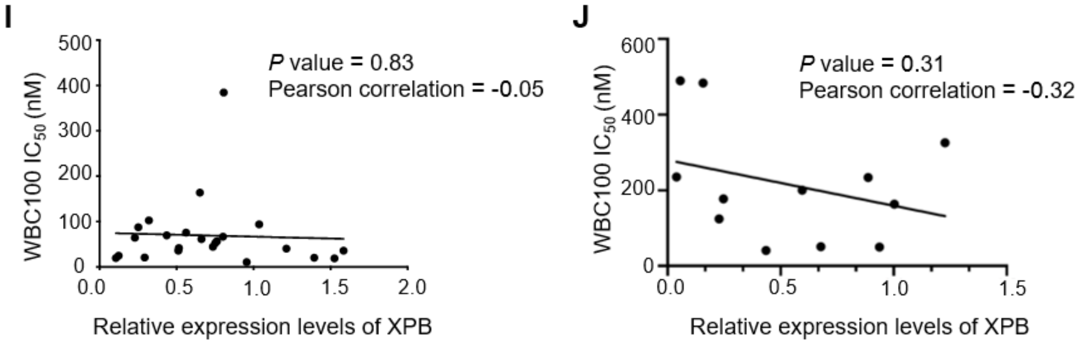

Figure S1. WBC100 is selectively toxic to c-Myc over-expressing cancer cells but sparing c-Myc-low normal cells. (**A, B**) The structure and formula of WBC100 (A) and its major metabolite (B). (**C**) Anti-proliferative activity of WBC100 against c-Myc over-expressing tumor cells (H9, MOLM-13, and Mia-paca2) and c-Myc-low normal cells (MRC-5, WI38, and L02) after treatment with WBC100 for 72 hours (n=3). (**D**) Western blot analysis of c-Myc protein levels in tumor and normal cells. (**E**) Western blot analysis of normal peripheral blood cells from 9 healthy adult donors. P: c-Myc over-expressing tumor cells as the positive control. (**F**) Anti-proliferative activity of WBC100 against normal peripheral blood cells after treatment with WBC100 for 72 hours (n=3). (**G**, **H**) Western blot analyses of c-Myc and XPB protein levels in 23 various hematological malignant cell lines (G) and 12 various solid tumor cell lines (H). (**I**, **J**) Correlation analyses between WBC100 anti-tumor activity and XPB levels in various hematological (I) and solid tumor (J) cell lines. The Pearson correlation coefficient and *P*-value calculated by Linear regression and correlation analysis using GraphPad are shown. All error bars represent means ± s.d. Statistical significance was determined by a two-tailed t-test.

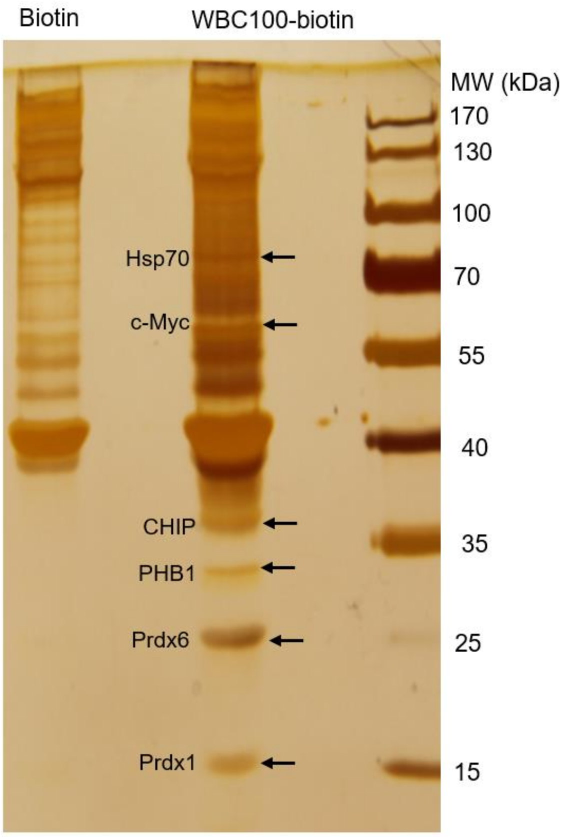

Figure S2. Identification of potential targets of WBC100 by Co-IP assay. HEK293T cell lysates were incubated with 5 μM WBC100-biotin or biotin as control. Proteins were then purified using streptavidin-conjugated magnetic beads, followed by SDS-PAGE analysis and silver-stained. The bands were retrieved and analyzed by mass spectrometry. Arrows indicated protein bands confirmed as Hsp70, c-Myc, E3 ligase CHIP, prohibitin-1 (PHB1), peroxiredoxin 6 (Prdx6), and peroxiredoxin 1(Prdx1).

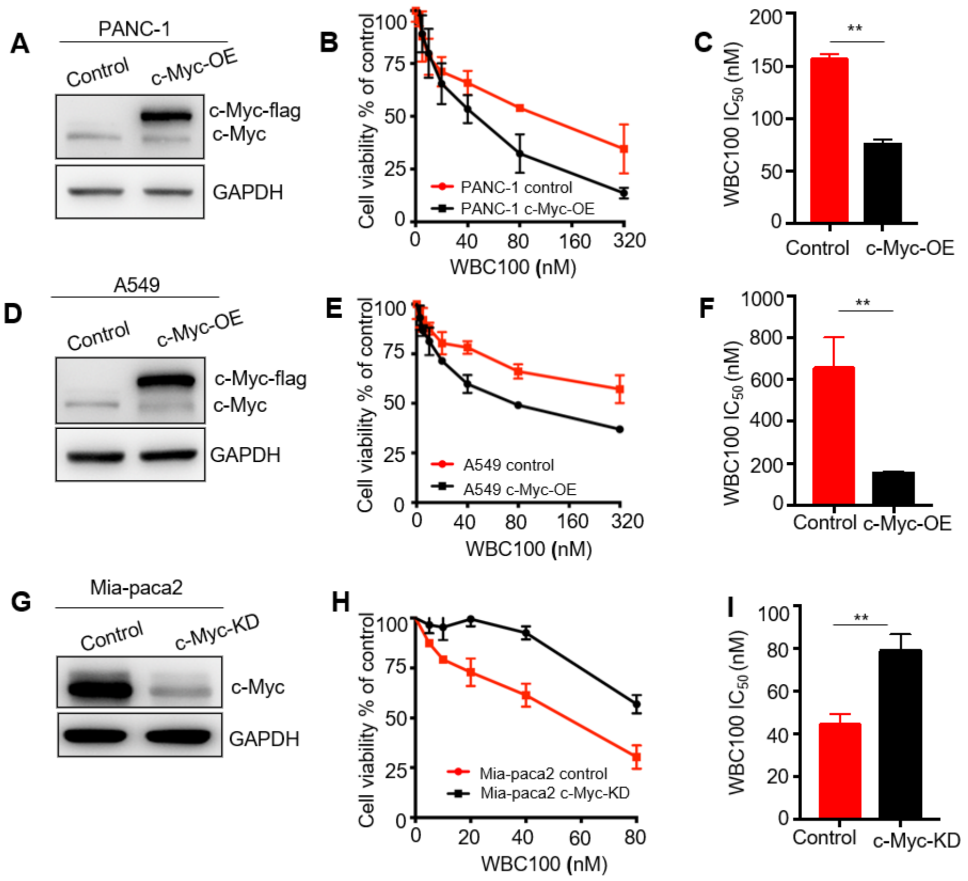

Figure S3. Correlation of wild-type c-Myc levels with WBC100 anti-tumor activity. (**A**) Western blot analysis of c-Myc in PANC-1 cells stably overexpressing Flag-tagged c-Myc or vector. c-Myc: endogenous c-Myc. (**B, C**) Sensitivity comparison of control and c-Myc overexpressing PANC-1 cells. Cells were treated with indicated concentrations of WBC100 for 96 hours. Representative results (B) and quantification (C) of WBC100 IC50 were shown (n=2). (**D**) Western blot analysis of c-Myc in A549 cells stably overexpressing Flag-tagged c-Myc or vector. (**E, F**) Sensitivity comparison of control and c-Myc overexpressing A549 cells. Cells were treated with indicated concentrations of WBC100 for 96 hours. Representative results (E) and quantification (F) of WBC100 IC50 were shown (n=3). (**G**) Western blot analysis of c-Myc knockdown by lentiviral shRNA in Mia-paca2 cells. (**H, I**) Sensitivity comparison of control and c-Myc knockdown Mia-paca2 cells. Cells were treated with indicated concentrations of WBC100 for 96 hours. Representative results (H) and quantification (I) of WBC100 IC50 were shown (n=3). All error bars represent means ± s.d. Two-tailed unpaired t-test, $^{**}P < 0.01$.

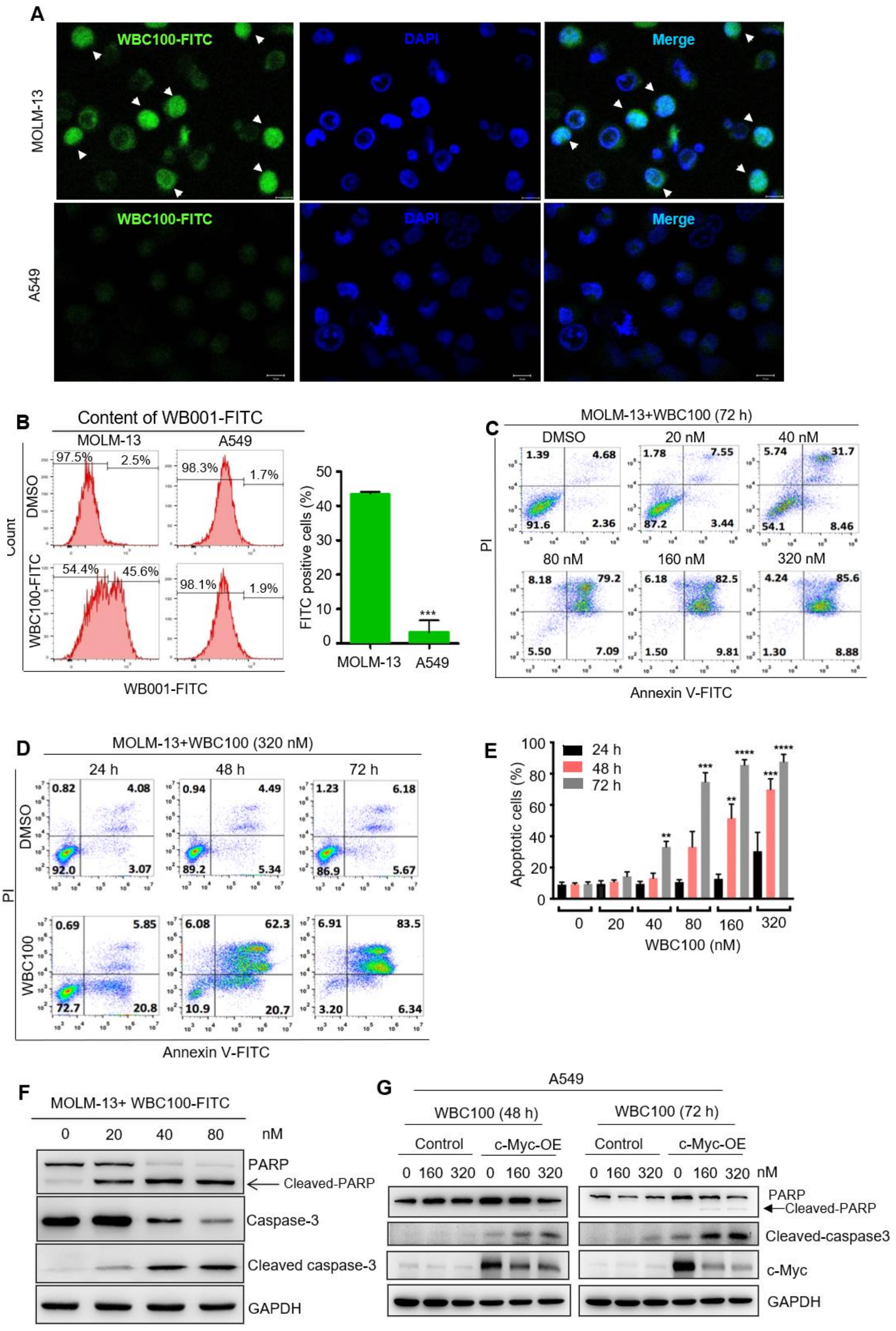
A
WBC100-FITC
DAPI
Merge
MOLM-13
A549
B
Content of WB001-FITC
MOLM-13
A549
DMSO
WBC100-FITC
Count
WB001-FITC
FITC positive cells (%)
C
MOLM-13+WBC100 (72 h)
DMSO
20 nM
40 nM
80 nM
160 nM
320 nM
PI
Annexin V-FITC
D
MOLM-13+WBC100 (320 nM)
24 h
48 h
72 h
E
Apoptotic cells (%)
WBC100 (nM)
F
MOLM-13+ WBC100-FITC
PARP
Cleaved-PARP
Caspase-3
Cleaved caspase-3
GAPDH
G
A549
WBC100 (48 h)
WBC100 (72 h)
Control
c-Myc-OE
Cleaved-caspase3
c-Myc

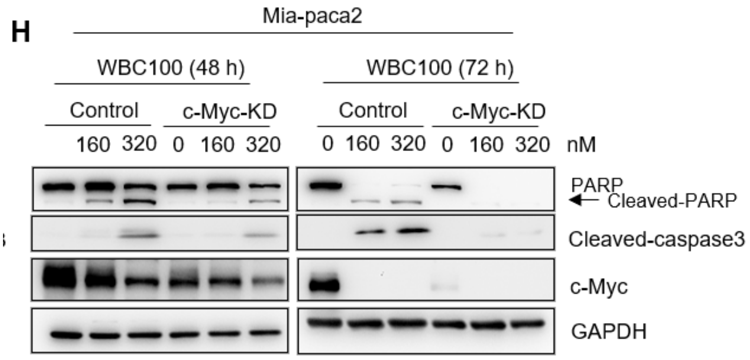

Figure S4. WBC100 accumulates in the nucleus and induces apoptosis of c-Myc over-expressing cancer cells in a c-Myc dependent way. (**A, B**) Subcellular distribution analysis of WBC100-FITC (arrowheads) (A) and WBC100-FITC content analysis (B) by flow cytometry in c-Myc over-expressing MOLM-13 and c-Myc-low A549 cells after treatment with 50 nM WBC100-FITC for 24 hours (n=3). Scale bars: 10 μm. (**C**) Apoptosis analysis in MOLM-13 treated with WBC100 for 72 hours at indicated concentrations by flow cytometry. Results were representative of three biological replicates. (**D**) Apoptosis analysis in MOLM-13 treated with WBC100 at 320 nM for indicated time points by flow cytometry. Results were representative of three biological replicates. (**E**) Quantification of apoptotic MOLM-13 cells treated with indicated concentrations of WBC100 for 24, 48, and 72 hours (n=3). (**F**) Western blot analysis of apoptosis-related proteins in MOLM-13 treated with indicated concentrations of WBC100-FITC for 24 hours. Arrows, proteolytic fragments of PARP (p89) (i.e., cleaved-PARP). (**G, H**) Western blot analysis of PARP, cleaved caspase3, and c-Myc in A549 c-Myc-OE (G) and Mia-paca2 c-Myc-KD (H) cells treated with indicated concentrations of WBC100 for 48 and 72 hours. All error bars represent means ± s.d. Two-tailed unpaired t-test, **P< 0.01, ***P< 0.001, ***P< 0.0001 .

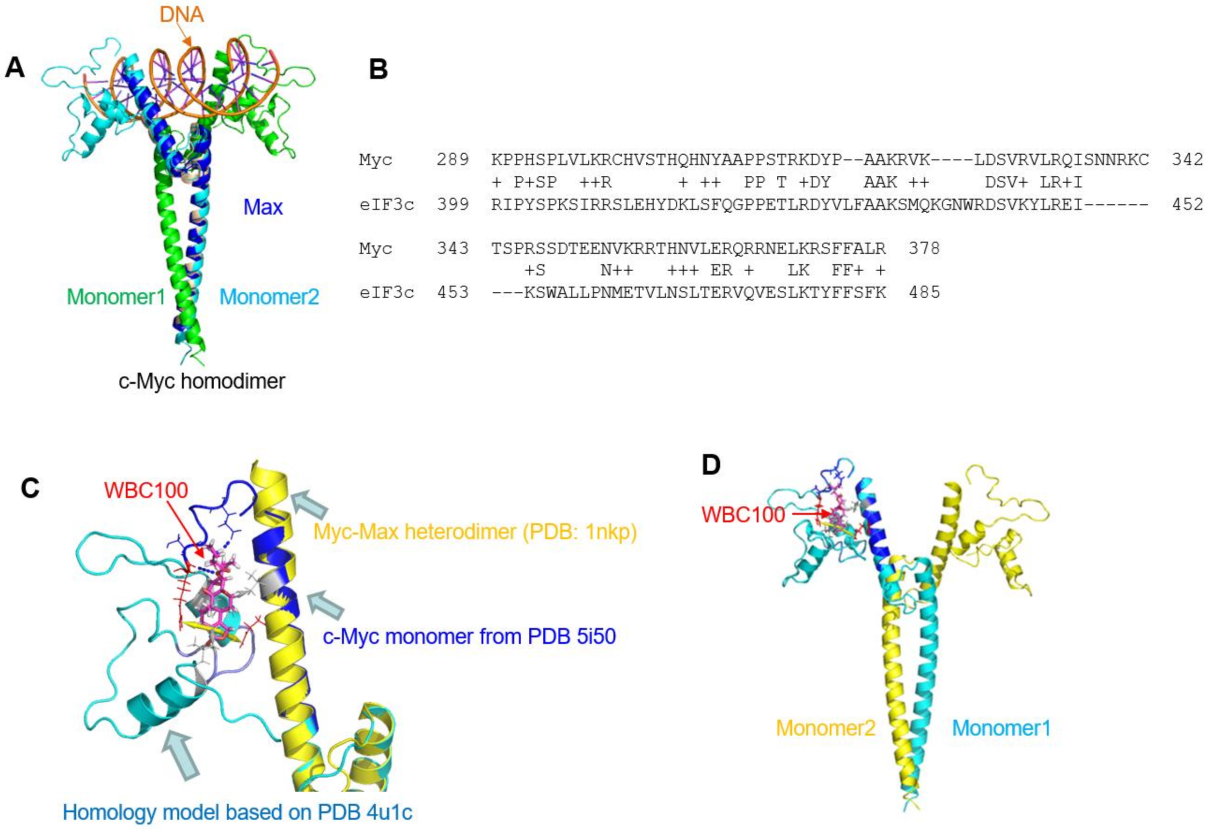

Figure S5. Binding model of WBC100 to c-Myc protein. (**A**) Model for c-Myc homodimer (green and cyan-colored) and superposed with Max protein (dark-blue colored from PDB id 6g6k) and DNA molecule (orange-colored from PDB id 5i50) to interact with c-Myc (green-colored). (**B**) Sequence alignment of c-Myc to eIF3c (PDB id 4u1c). The sequence identity is 27%. (**C**) A homology model to dock WBC100 by merging two regions of c-Myc: one region of 350-439aa by using the structure c-Myc monomer part of PDB 5i50 (blue-colored), and the other region of 289-349aa by using homology structure of eIF3c (PDB 4u1c, cyan-colored). The Myc-Max heterodimer form PDB 1nkp (yellow-colored) was used to show its similarity to c-Myc monomer part of PDB 5i50 (see the overlaps between blue-colored and yellow-colored). (**D**) WBC100 compound binds at a region that is not dimer interaction region.

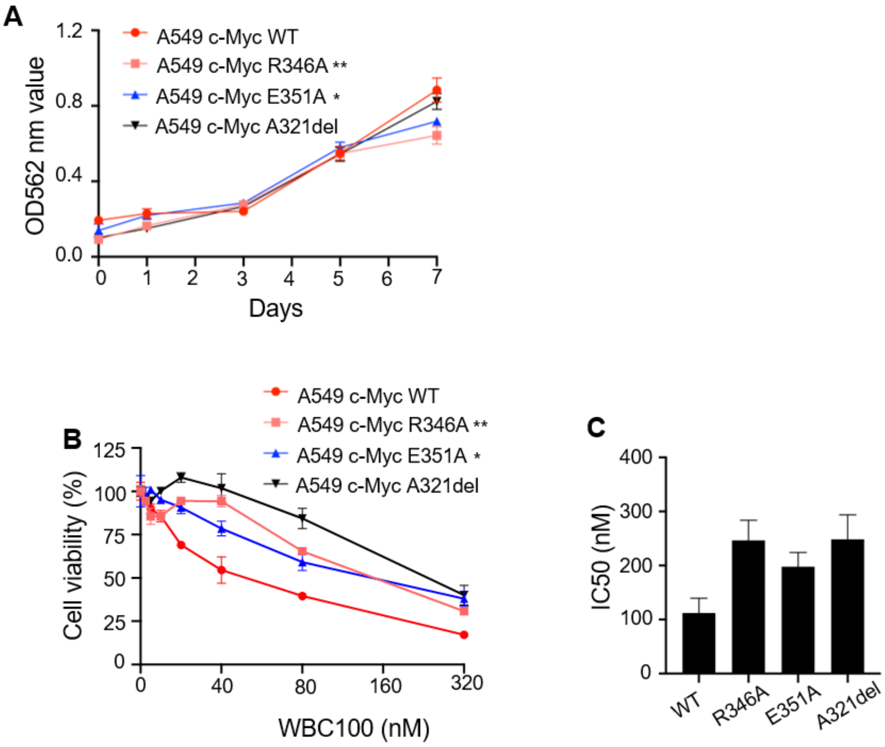

Figure S6. Effects of c-Myc-mutations on the proliferation of A549 cells and WBC100-mediated anti-tumor activity. (**A**) Comparisons of A549 cell proliferation activity among wild-type c-Myc or c-Myc mutants (R346A, E351A, and A321del), assessed by MTT (n=2). (**B, C**) Effects of c-Myc mutations on WBC100-induced anti-tumor activity. Cells were treated with indicated concentrations of WBC100 for 96 hours. Representative results (B) and quantification (C) of WBC100 IC50 were shown (n=2). All error bars represent means ± s.d. Two-tailed unpaired t-test, *P< 0.05, **P< 0.01.

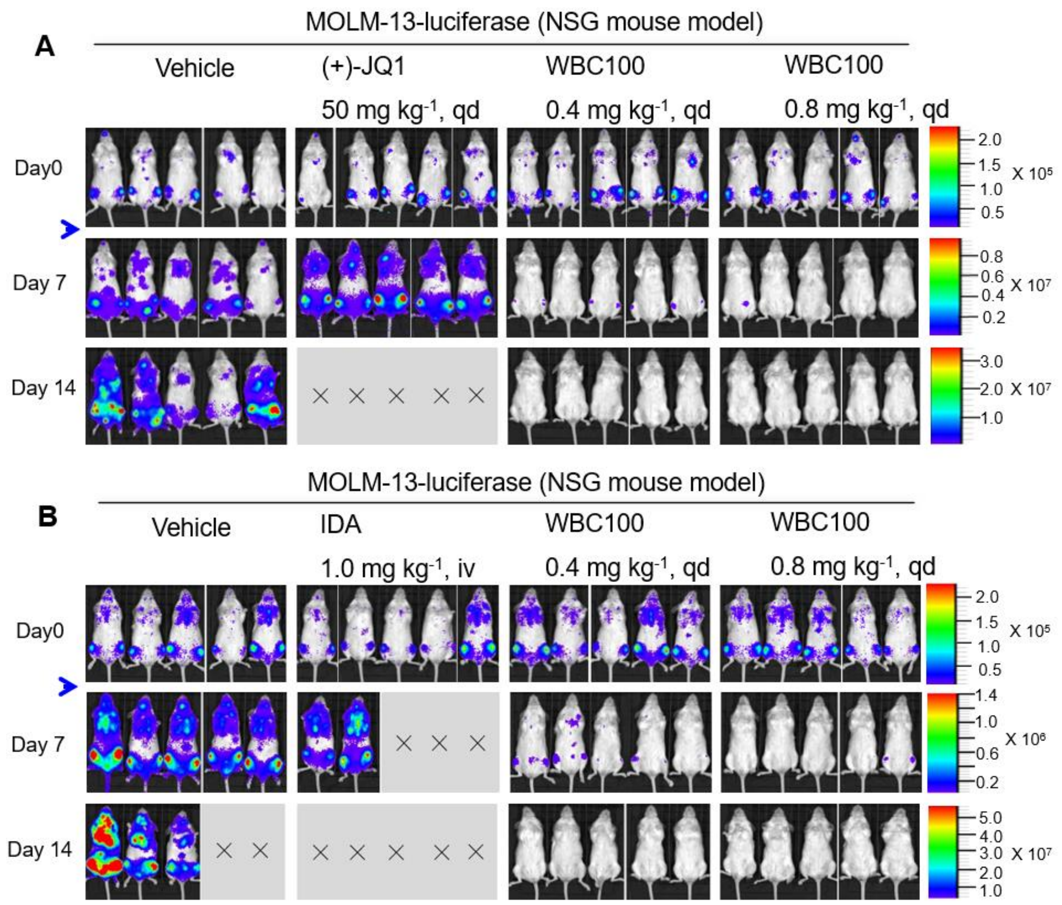

Figure S7. Anti-tumor activity of WBC100 is superior to (+)-JQ1 and IDA in c-Myc over-expressing refractory AML in NSG mice. (**A**) Bioluminescence images of NSG mice bearing MOLM-13-luciferase cells at indicated time points after treatment with vehicle, (+)-JQ1 and WBC100 (n=5 per group). (**B**) Bioluminescence images of NSG mice bearing MOLM-13-luciferase cells at indicated time points after treatment with vehicle, IDA (idarubicin), and WBC100 (n=5 per group). Arrows indicated the beginning of treatment.

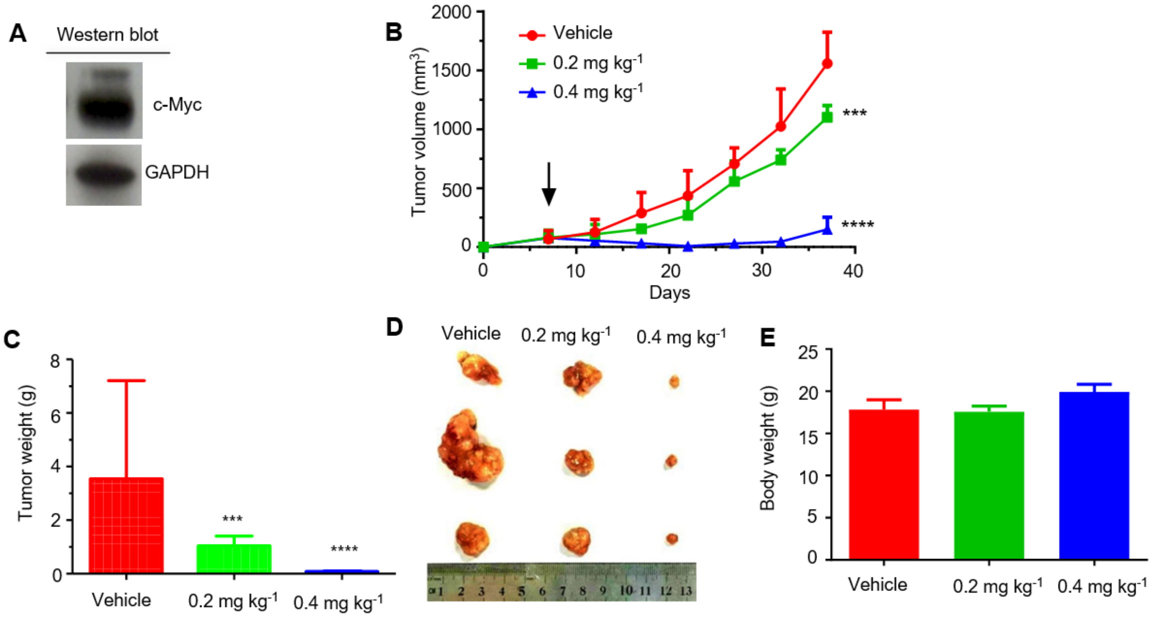

Figure S8. WBC100 potently regresses c-Myc over-expressing human gastric cancer in vivo. (**A**) Western blot analysis of c-Myc protein in MGC-803 cells. (**B**) Average tumor volumes of MGC-803 xenografts in NOD/SCID mice (n=3 per group) treated with vehicle or WBC100 at indicated time points. Arrows indicated the beginning of treatment. (**C**) Tumor weight comparison between indicated treatment groups (n=3 per group). (**D**) Gross appearances of tumors from mice of each treatment group. (**E**) Mouse body weight comparison between indicated treatment groups.All error bars represent means ± s.e.m.***P*< 0.01, ****P*< 0.001, *****P*< 0.0001.

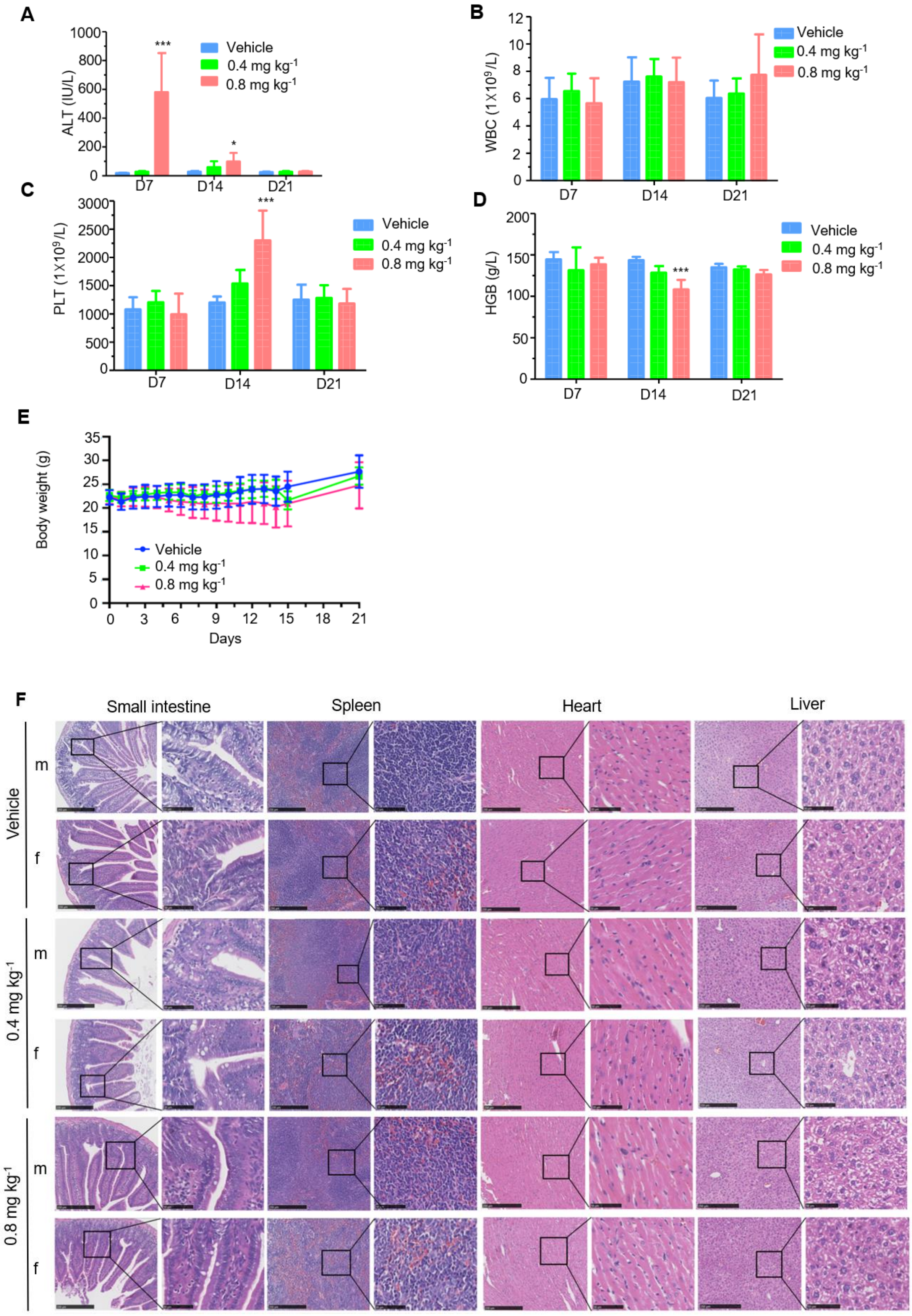
A
ALT (IU/L)
***
*
Vehicle
0.4 mg kg-1
0.8 mg kg-1
D7
D14
D21
B
WBC (1X10^9/L)
C
PLT (1X10^9/L)
***
D
HGB (g/L)
***
E
Body weight (g)
Days
F
Small intestine
Spleen
Heart
Liver
Vehicle
0.4 mg kg-1
0.8 mg kg-1
m
f

Figure S9. Effects of WBC100 on blood cell counts, liver enzyme ALT, and body weight of healthy mice. (**A-E**) Healthy normal mice (n=6) were treated with continue oral administration (twice daily) of WBC100. ALT (A), WBC (B), PTL (C), HGB (D) and body weight (E) were evaluated. ALT: alanine amino transferase, WBC: white blood cell, PLT: platelet, HGB: hemoglobin. (**F**) Representative gross histology images of the liver, small intestine, spleen, and heart in healthy normal mice treated with WBC100 for safety evaluation. f: female. m: male.Scale bars: 250 μm (20 ×), 50 μm (40 ×). All error bars represent means ± s.e.m. *P<0.05, ***P<0.001.
